# CryoSegNet: accurate cryo-EM protein particle picking by integrating the foundational AI image segmentation model and attention-gated U-Net

**DOI:** 10.1093/bib/bbae282

**Published:** 2024-06-11

**Authors:** Rajan Gyawali, Ashwin Dhakal, Liguo Wang, Jianlin Cheng

**Affiliations:** Department of Electrical Engineering and Computer Science, University of Missouri, Columbia, MO 65211, United States; NextGen Precision Health, University of Missouri, Columbia, MO 65211, United States; Department of Electrical Engineering and Computer Science, University of Missouri, Columbia, MO 65211, United States; NextGen Precision Health, University of Missouri, Columbia, MO 65211, United States; Laboratory for BioMolecular Structure (LBMS), Brookhaven National Laboratory, Upton, NY 11973, United States; Department of Electrical Engineering and Computer Science, University of Missouri, Columbia, MO 65211, United States; NextGen Precision Health, University of Missouri, Columbia, MO 65211, United States

**Keywords:** Cryo-EM, protein particle picking, image segmentation, machine learning, attention-gated U-Net, Segment Anything Model (SAM)

## Abstract

Picking protein particles in cryo-electron microscopy (cryo-EM) micrographs is a crucial step in the cryo-EM-based structure determination. However, existing methods trained on a limited amount of cryo-EM data still cannot accurately pick protein particles from noisy cryo-EM images. The general foundational artificial intelligence–based image segmentation model such as Meta’s Segment Anything Model (SAM) cannot segment protein particles well because their training data do not include cryo-EM images. Here, we present a novel approach (CryoSegNet) of integrating an attention-gated U-shape network (U-Net) specially designed and trained for cryo-EM particle picking and the SAM. The U-Net is first trained on a large cryo-EM image dataset and then used to generate input from original cryo-EM images for SAM to make particle pickings. CryoSegNet shows both high precision and recall in segmenting protein particles from cryo-EM micrographs, irrespective of protein type, shape and size. On several independent datasets of various protein types, CryoSegNet outperforms two top machine learning particle pickers crYOLO and Topaz as well as SAM itself. The average resolution of density maps reconstructed from the particles picked by CryoSegNet is 3.33 Å, 7% better than 3.58 Å of Topaz and 14% better than 3.87 Å of crYOLO. It is publicly available at https://github.com/jianlin-cheng/CryoSegNet

## Introduction

Protein structure determination is a significant area of research in the field of structural biology and bioinformatics, enabling researchers to understand the roles of proteins in various biological processes [1]. This structural insight is important for studying the interaction of proteins with other molecules in the cellular processes. It is useful for finding the potential binding sites for drug molecules to act on to modulate the function of proteins [2, 3]. Further, many diseases are the result of protein misfolding and aggregation. Thus, it is imperative to determine the protein structure for understanding protein function and interaction, studying their roles in the diseases and accelerating the design of drugs.

X-ray crystallography, nuclear magnetic resonance (NMR) and cryo-EM [4, 5] are three main experimental techniques to determine protein structures. Among them, cryo-EM is the cutting-edge technique for solving the structure of large protein complexes. With advancements in electron microscope and detector devices, cryo-EM has revolutionized the field of structural biology and enabled the determination of very large protein complex structures at near atomic resolution that other experimental techniques cannot handle.

The cryo-EM-based structure determination process [6, 7] involves sample preparation with vitreous ice, imaging them with electron dose from the microscope to generate two-dimensional (2D) projections of the samples at different orientations, followed by protein particle picking in cryo-EM micrographs (images). Once the particles are picked and extracted, the single particle analysis is employed to determine the three-dimensional (3D) structure of the specimen.

Particle picking in cryo-EM micrographs has posed significant challenges due to the low contrast of micrographs with a low signal-to-noise ratio (SNR) caused by using limited electron dose during imaging process. Further, the prevalence of ice contamination, carbon edges, protein aggregates and deformed particles have further complicated the particle picking. Reconstructing a 3D protein structure from cryo-EM micrographs requires thousands of extracted particles of good quality, and therefore, it is important to pick protein particles accurately and automatically, releasing the burden of human intervention and reducing the bias and inconsistency associated with manual particle picking.

With advancements in hardware and software tools [8–12], numerous semi-automated or automated approaches varying from traditional computational methods to modern deep learning techniques have been proposed to streamline the cryo-EM processing and particle picking. Conventional computer vision methods like edge detection, blob detection and template matching [4] are still widely used for particle picking. However, due to the low SNR of cryo-EM micrographs, these techniques are susceptible to picking ice patches, carbon areas and aggregated particles, resulting in a high number of false positives. RELION [11] leverages a regularized likelihood optimization technique and utilizes the template-based and blob-based picking [13] approaches. In the template-based approach, an initial set of 2D templates are generated from the manually picked particles, which are used to correlate with the different regions of micrographs to extract similar patches. This approach is highly sensitive to noise and may introduce significant bias. Similarly, in the blob-based picking, the regions of high intensity and local maxima are extracted from cryo-EM micrographs using Laplacian of Gaussian. This method is useful if the particles have significant contrast difference with the background of the micrographs and all the particles within the micrograph are of similar shape and size. If the particles are of different conformations and size, this method faces a lot of difficulty in picking the true protein particles. Other conventional tools like EMAN2 [10], SPIDER [14] and XMIPP [15] utilizing similar computer vision approaches require a lot of manual intervention, computational resources, memory and human time and face significant challenges of filtering out false positives.

Recent advancements in machine learning, particularly deep learning, have shown great potential for particle picking. Several machine learning approaches have been put forth to automate the particle picking process and reduce the number of false positives. Notable approaches include APPLE picker [16], crYOLO [17], PIXER [18], WARP [19], Topaz [20], CASSPER [21], Deep Picker [22], AutoCryoPicker [23], DeepCryoPicker [24], DRPnet [25] and CryoTransformer [26]. They utilize either convolutional neural networks or unsupervised learning algorithms like clustering. Nevertheless, these methods typically underwent training with a limited set of micrographs. For instance, crYOLO was trained with only 840 micrographs. Consequently, they may struggle to generalize effectively to diverse protein types characterized by irregular and complex shapes, as well as heterogenous conformations. They often overlook the diversity of the proteins and are usually evaluated on one or a few simple datasets like Apoferritin and Keyhole Limpet Hemocyanin (KLH) due to lack of manually annotated particle data. Among these methods, crYOLO and Topaz are most widely used. CrYOLO utilizes the You Only Look Once (YOLO), an object detection algorithm [27] trained on cryo-EM micrographs, and Topaz employs positive-unlabeled convolutional neural networks [20] for particle picking. While both approaches have demonstrated significant potential in automating particle picking, their training has been based on a relatively small number of micrographs. CrYOLO often misses many true protein particles while Topaz picks too many particles including false positives and duplicates. The large number of particles picked by Topaz also causes difficulty in storing and processing the extracted particles required for the down-stream processing steps. As a result, the potential of deep learning for particle picking has not yet been fully harnessed, and the cryo-EM community still needs to mostly rely on traditional semi-automated methods like template-based picking in tools like RELION and CryoSPARC to perform particle picking, which are time consuming and error-prone.

Two recent developments provide good opportunities to further improve automated particle picking. The first is the recent creation of a large, labeled protein particle dataset—CryoPPP [4] from the Electron Microscopy Public Image Archive (EMPIAR) [28], which enables the development and training of sophisticated deep learning methods for particle picking. The second one is the availability of large foundational artificial intelligence (AI) image segmentation models such as Meta’s Segment Anything Model (SAM) [29] that may be used to segment objects in images. However, a direct application of SAM to cryo-EM images can segment few particles because cryo-EM images are very different from the image data used to train SAM. Moreover, a simple retraining of SAM on cryo-EM images only yielded somewhat improved but still unsatisfactory results.

To leverage the opportunities and address the challenges above, we first designed a specialized U-Net architecture [30] with the inclusion of attention gates in each decoder block and trained it on the CryoPPP dataset to pick protein particles. After training, the attention-gated U-Net is applied to any cryo-EM micrograph to generate a segmentation map as input for SAM’s automatic mask generator [29] for accurately localizing protein particles in the cryo-EM micrograph. This segmentation network of integrating the specialized U-Net architecture and SAM for particle picking (called CryoSegNet) performs better than the two most popular AI-based pickers crYOLO and Topaz, the recently developed CryoTransformer and other AI pickers like CASSPER and Deep Picker in terms of both the accuracy of particle picking and the resolution of 3D protein density maps reconstructed from picked particles. Particularly, CryoSegNet substantially increases the resolution of density maps constructed from picked particles over crYOLO and Topaz, making it a useful tool for generating more accurate protein structures from both existing and new cryo-EM image data.

## Results and discussion

### Combining the specialized attention-gated U-Net trained on cryo-EM images with the general foundational Segment Anything Model for particle picking

**Figure 1:**
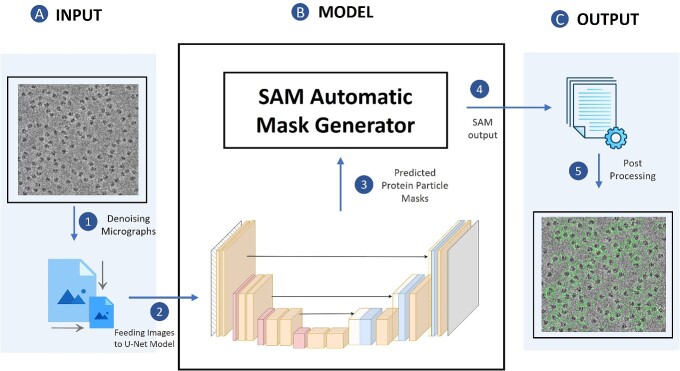
The process of particle picking with CryoSegNet. (**A**) An input micrograph is first denoised and then sent to the U-net model. (**B**) U-Net model outputs a segmentation mask for each micrograph that is fed to SAM automatic mask generator for predicting the bounding boxes of protein particles. (**C**) The output generated by SAM is further processed based on thresholding the prediction confidence scores to filter out some false particles to generate the final output of picked particles stored in .star files.

Figure 1 illustrates the process of particle picking from cryo-EM micrographs using CryoSegNet. A cryo-EM micrograph is first denoised by the image processing techniques [23, 31, 32]. The denoised micrograph is then used as input for an attention-gated U-Net trained on a comprehensive and diverse dataset consisting of thousands of manually labeled cryo-EM micrographs of 22 diverse protein types to pick particles to generate a segmentation map, which is used as input for Segment Anything Model (SAM) to generate a mask map with identified particles. The particles in the mask map are further post-processed (e.g. combined or filtered) by a post-processing module to generate the final output containing the picked particles. The final output includes the protein particle coordinates in the form of .star files, which are compatible with widely used tools like RELION [11] and CryoSPARC [12] and can be directly used by them to generate 3D protein density maps.

After CryoSegNet was trained and validated on the training/validation, we blindly benchmarked it on a test dataset consisting of thousands of labeled cryo-EM micrographs of seven different protein types from the CryoPPP [4] dataset. The particles picked by CryoSegNet were compared with the ground truth coordinates of the expert-labeled particles. The standard image segmentation metrics including precision, recall, F1-score (i.e. $\frac{precision\times recall}{\left( precsion+ recall\right)/2}$) and Dice score [33] of particle picking made by CryoSegNet were calculated to evaluate its performance. Dice score is used to evaluate the similarity between predicted segmentation masks and ground truth masks. It ranges from 0 (zero overlap) to 1 (perfect overlap). Furthermore, as an ultimate test, we constructed 3D density maps for each protein from the particles picked by CryoSegNet, Deep Picker, crYOLO, Topaz, CASSPER and CryoTransformer, respectively, and compared the resolution of the reconstructed density maps. The detailed results are reported in the subsections below.

### The performance of particle picking on the CryoPPP test dataset in terms of image segmentation metrics

The number of cryo-EM micrographs and labeled particles for each of the seven different types of proteins in the CryoPPP test dataset is reported in Table 1. There are 1,879 labeled cryo-EM images and 401,263 labeled particles in total, which form the largest test dataset for evaluating particle picking methods to date. To fairly compare the six AI methods: Deep Picker, CrYOLO, Topaz, CASSPER, CryoTransformer and CryoSegNet, we trained and tested all these methods with the same set of training, validation and test data.

**Table 1:** Evaluation results on the CryoPPP test dataset. The EMPIAR ID of the cryo-EM image set for each of the seven test proteins is listed in column 1. The type of each protein, number of cryo-EM images and number of labeled particles are reported in columns 2–4. The precision, recall, F1-score and Dice score are reported in the other columns. Bold font denotes the best average score of each metric

EMPIAR ID	Type of protein	Num. of labeled images	Num. of labeled particles	Metrics	Template-based	Deep Picker	CrYOLO	Topaz	CASSPER	CryoTransformer	CryoSegNet
10028 [34]	Ribosome (80S)	300	26,391	Precision	0.845	0.848	0.807	0.696	0.877	0.751	0.833
				Recall	0.935	0.920	0.941	0.937	0.591	0.832	0.944
				F1-Score	0.888	0.883	0.869	0.799	0.706	0.789	0.885
				Dice Score	0.872	0.861	0.863	0.786	0.668	0.826	0.859
10081 [35]	Transport	300	39,352	Precision	0.853	0.868	0.822	0.732	0.915	0.860	0.835
				Recall	0.900	0.900	0.884	0.872	0.796	0.889	0.922
				F1-Score	0.876	0.883	0.852	0.796	0.851	0.874	0.876
				Dice Score	0.877	0.822	0.822	0.758	0.834	0.823	0.876
10345 [36]	Signaling	295	15,894	Precision	0.743	0.912	0.648	0.544	0.598	0.744	0.746
				Recall	0.924	0.504	0.665	0.805	0.716	0.864	0.920
				F1-Score	0.824	0.649	0.656	0.650	0.652	0.799	0.824
				Dice Score	0.707	0.578	0.452	0.507	0.532	0.684	0.743
11056 [37]	Transport	305	125,908	Precision	0.717	0.717	0.726	0.764	0.768	0.853	0.757
				Recall	0.306	0.306	0.780	0.909	0.292	0.683	0.687
				F1-Score	0.429	0.429	0.752	0.830	0.423	0.758	0.720
				Dice Score	0.401	0.401	0.718	0.778	0.398	0.679	0.663
10532 [38]	Viral	300	87,933	Precision	0.790	0.621	0.756	0.732	0.848	0.813	0.796
				Recall	0.716	0.682	0.774	0.939	0.385	0.665	0.628
				F1-Score	0.751	0.650	0.765	0.823	0.530	0.732	0.702
				Dice Score	0.743	0.432	0.724	0.788	0.519	0.614	0.649
10093 [39]	Membrane	295	56,394	Precision	0.672	0.756	0.623	0.610	0.632	0.560	0.716
				Recall	0.613	0.349	0.744	0.216	0.596	0.689	0.515
				F1-Score	0.641	0.478	0.678	0.319	0.613	0.618	0.600
				Dice Score	0.568	0.437	0.641	0.279	0.513	0.600	0.537
10017 [40]	β-Galactosidase	84	49,391	Precision	0.814	0.795	0.824	0.847	0.860	0.745	0.859
				Recall	0.822	0.691	0.588	0.936	0.770	0.587	0.616
				F1-Score	0.818	0.739	0.686	0.889	0.813	0.657	0.718
				Dice Score	0.813	0.764	0.663	0.886	0.808	0.623	0.703
**Average**				Precision	0.776	0.788	0.744	0.704	0.785	0.761	**0.792**
				Recall	0.745	0.622	0.768	**0.802**	0.592	0.744	0.747
				F1-Score	0.747	0.673	0.751	0.729	0.655	0.747	**0.761**
				Dice Score	0.712	0.614	0.698	0.683	0.610	0.693	**0.719**

Deep Picker was trained with default parameters in CryoSPARC, CrYOLO with ‘PhosaurusNet’ architecture and Topaz with ‘ResNet16’ architecture. CASSPER and CryoTransformer were trained with their default parameters. The details of parameters used in training of CrYOLO and Topaz can be found in Supplementary Note S1. The per-protein and average precision, recall, F1-score and Dice score of all the AI methods and the template-based picking on the dataset are summarized in Table 1. The average precision, recall, F1-score and Dice score of CryoSegNet are 0.792, 0.747, 0.761 and 0.719, respectively, while for CrYOLO, they are 0.744, 0.768, 0.751 and 0.698. Topaz has an average precision, recall, F1-score and Dice score of 0.704, 0.802, 0.729 and 0.683, respectively. For CryoTransformer, the average precision, recall, F1-score and Dice score are 0.761, 0.744, 0.747 and 0.693, respectively. Among these methods, CryoSegNet has the highest F1-score, precision and Dice score, while Topaz has the highest recall. The higher F1-score of 0.761 for CryoSegNet, in contrast to 0.729 for Topaz, 0.747 for CryoTransformer and 0.751 for CrYOLO, indicates that CryoSegNet is a more balanced particle picker than Topaz, CryoTransformer and CrYOLO, considering both sensitivity (recall) and specificity (precision). The template-based picking also shows relatively good performance, while Deep Picker and CASSPER performs substantially worse than CryoSegNet in terms of F1-score and Dice score.

**Figure 2:**
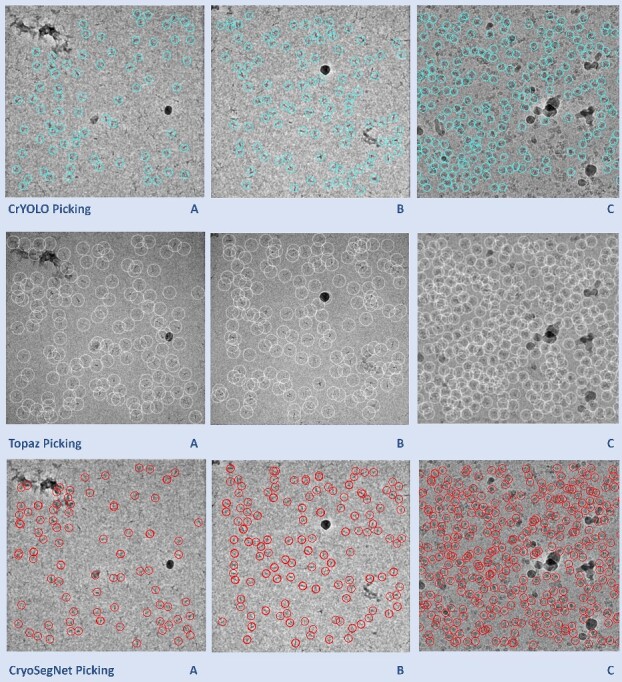
Comparison of particle picking by crYOLO, Topaz and CryoSegNet on three cryo-EM micrographs of two protein types (EMPIAR ID 10345 and EMPIAR ID 11056). (**A**) Topaz picks ice patches and more particles in the contaminated regions than CryoSegNet while crYOLO picks few particles (EMPIAR ID 10345). (**B**) Topaz picks more false positives (particularly the ones on the black ice patch) compared to CryoSegNet (EMPIAR ID 10345). (**C**) CryoSegNet picks a zero to small number of particles in undesired (carbon or ice) regions (black holes) of the micrograph (EMPIAR ID 11056), while Topaz picks some false particles in the regions.

Moreover, we compared the predictions made by the crYOLO, Topaz and CryoSegNet for some individual micrographs to study their characteristics. Figure 2 illustrates the typical disparities in particle picking among crYOLO, Topaz and CryoSegNet on three individual cryo-EM micrographs of two protein types (EMPIAR ID 10345 and EMPIAR ID 11056). CrYOLO tends to pick fewer protein particles, thereby discarding many true particles. Topaz, when using with default parameters, picks an excessive number of true particles with a lot of overlaps (redundancy) as well as false particles within carbon edges and ice patches that can cause a serious difficulty for the 3D reconstruction of density maps from the picked particles. The storage requirement for processing the redundant particles from Topaz for 3D reconstruction is substantial. In contrast, CryoSegNet usually picks most true protein particles while selecting only a small number of false positives, minimizing the number of redundant/duplicated/overlapped particles and largely excluding false particles in the carbon edges and ice patches.

We also compare the precision, recall, F1-score and Dice score of the output of each of the three prediction modules of CryoSegNet: (1) the attention-gated U-Net, (2) the SAM and (3) the postprocessing module (Supplementary Table S1). At the end of each subsequent module, the F1-scores are computed, revealing higher values for SAM (0.768) and the postprocessing module (0.761) in comparison to U-Net (0.71). This indicates that the performance is improved by incorporating SAM into the output of U-Net. Interestingly, applying the SAM module to the output of the U-Net substantially increases the recall from 0.739 to 0.820, while decreasing the precision from 0.747 to 0.729. Adding the post-processing on top of the SAM output increases the precision from 0.729 to 0.792, while decreasing the recall from 0.820 to 0.747. At the end, the precision of the final output of CryoSegNet (e.g. the output of the post-processing module) is substantially higher than the U-Net (0.792 versus 0.747), while its recall is slightly higher than the U-Net (0.747 versus 0.739), resulting in a higher F1-score (0.761 versus 0.71). The results show that the three prediction steps of CryoSegNet complement each other, leading to the balanced performance.

### The performance of particle picking in terms of the resolution of 3D density maps reconstructed from picked particles

The F1-score, precision and recall of particle picking can measure the accuracy of a machine learning method discriminating particles from non-particles, but they do not directly measure the quality of the density maps of proteins reconstructed from the picked particles, which are the end products concerning users most. Reconstructing 3D density maps from picked particles involves very complex algorithms of converting 2D particle images to 3D density maps, whose performance depends on many factors such as the number of true particles, the uniqueness of true particles capturing different orientations (views) of protein structure and the severity of false particles that cannot be simply measured by a single score such as F-measure, precision and recall. Therefore, as an ultimate test, we compare CryoSegNet, Topaz and crYOLO in terms of the resolution of 3D density maps reconstructed from picked particles on CryoPPP test dataset.

#### The comparison of the resolution of the density maps reconstructed from the particles picked by six AI methods and the template-based picking on CryoPPP test dataset

For each protein type in the test dataset, we generate star files containing particles picked by a method, which are then imported into CryoSPARC for 3D *ab initio* reconstruction of density maps and homogenous refinement [12]. In the context of *ab initio* reconstruction, we reconstruct a 3D density map from only a set of particles without using any initial structural model or starting structure as input. Homogeneous refinement is employed to rectify higher-order aberrations and to refine particle defocus caused by factors such as beam tilt, spherical aberration and other optical issues. We compare the 3D resolution of the density maps reconstructed from the particles picked by the template-based picking, Deep Picker, crYOLO, Topaz, CASSPER, CryoTransformer and CryoSegNet. Results are computed both with and without considering the best 2D templates from the Select2D job [12] in CryoSPARC. Select2D is a process used by CryoSPARC internally to filter out low-quality/false particles provided by users before the density map reconstruction.

The experiments were conducted across three trials with random seed initialization, and the average resolution was considered for comparison. The summary results of these methods on the micrographs in CryoPPP test dataset are presented in Table 2, while the detailed trial results can be found in Supplementary Table S2. The resolution of both CryoSegNet and Topaz is higher than crYOLO on six out of seven protein types. CryoSegNet has a higher resolution than all the other methods on four out of seven protein types and the same best performance on one protein (EMPIAR ID 10532) with CryoTransformer. CrYOLO yields the highest resolution for EMPIAR 10017 and the template-based picking provides the best resolution for EMPIAR ID 10093. The average resolution of CryoSegNet with Select 2D is 4.98 Å, better than 5.19 Å of the template-based picking, 6.71 Å of Deep Picker, 5.41 Å of CrYOLO, 5.19 Å of Topaz, 5.77 Å of CASSPER and 5.65 Å of CryoTransformer. Also, on all seven protein types, CryoTransformer picked most particles (96,668 on average) followed by Topaz (43,842 on average) and template-based picking (39,673 on average) while CryoSegNet (32,321 on average) and crYOLO (33,401 on average) picked a similar number of particles, indicating that the quality of density maps does not fully depend on the number of picked particles. Further, Deep Picker (18,940 on average) and CASSPER (26,829 on average) picked a fewer number of particles. This result can be largely explained by the observation that Topaz identifies many particles with some redundancy/overlap, Deep Picker and CASSPER miss many true particles and CryoSegNet picks most true particles with little redundancy.

**Table 2:** Comparison of CryoSegNet with the template-based picking, Deep Picker, crYOLO, topaz, CASSPER and CryoTransformer in terms of the resolution of 3D density maps on CryoPPP test dataset. Bold font denotes the highest resolution

EMPIAR ID	Number of Particles							Average Resolution (Å)						
	Template-based	Deep Picker	CrYOLO	Topaz	CASSPER	CryoTransformer	CryoSegNet	Template-based	Deep Picker	CrYOLO	Topaz	CASSPER	CryoTransformer	CryoSegNet
10028	32,183	30,242	31,699	35,514	15,637	40,488	45,218	4.12	4.09	4.11	3.97	4.43	3.86	**2.72**
10081	41,569	28,209	36,821	37,808	27,299	88,632	44,819	5.26	6.05	5.38	5.10	5.79	5.47	**4.18**
10345	14,353	2,470	11,369	21,343	9,876	105,739	15,209	4.07	9.13	3.94	3.66	5.14	6.43	**2.89**
11056	53,190	17,124	43,599	66,651	34,860	98,193	53,073	8.12	9.65	8.54	8.06	8.47	7.42	**7.17**
10532	43,662	28,711	29,434	38,372	29,290	148,345	30,155	3.93	4.92	4.10	4.27	3.96	**3.92**	**3.92**
10093	42,986	2,360	33,183	61,698	32,383	151,545	27,745	**5.85**	7.50	6.93	6.15	7.27	6.86	7.06
10017	49,770	23,462	47,704	45,511	38,460	43,735	10,026	5.00	5.63	**4.87**	5.09	5.33	5.61	6.91
**Average**	39,673	18,940	33,401	43,842	26,829	96,668	32,321	5.19	6.71	5.41	5.19	5.77	5.65	**4.98**

Moreover, applying Select 2D to the density map reconstruction improves the resolution of all these methods. It is worth noting that, even though the results in Table 2 were obtained from particles picked from at most 305 micrographs for each protein type in CryoPPP test dataset, the resolution of CryoSegNet for some protein types is high. For instance, on two protein types (EMPIAR ID 10028 and 10345), the resolution of CryoSegNet, after removing some false positives by Select 2D, is below 3 Å.

#### The comparison of resolution of 3D density maps reconstructed from all cryo-EM micrographs of five protein types in EMPIAR

In addition to evaluating the on the test dataset from CryoPPP that has only approximately 300 micrographs for each protein type (see Table 1), we extended the assessment of the methods to the complete set of micrographs available on the EMPIAR website for five different protein types in CryoPPP test dataset (Table 3) to gauge the resolution that they can achieve in a real-world setting. CryoSegNet substantially outperform other methods on most protein types and on average.

**Table 3:** Comparison of 3D resolution of on the full set of micrographs of five protein types. The last column lists the resolution of the density maps built by their original authors as a reference

EMPIAR ID	Number of particles							Average resolution (Å)							Original EMPIAR Resolution (Å)
	Template-based	Deep Picker	CrYOLO	Topaz	CASSPER	CryoTransformer	CryoSegNet	Template-based	Deep Picker	CrYOLO	Topaz	CASSPER	CryoTransformer	CryoSegNet	
10028	60,901	43,027	63,562	96,352	29,906	81,172	92,532	3.97	4.09	3.96	**2.72**	4.17	3.74	**2.72**	3.2
10345	78,835	8,399	40,047	87,472	56,728	111,375	73,377	3.51	4.21	3.54	3.46	4.03	3.48	**2.69**	3.51
10081	134,603	96,322	123,963	130,941	115,297	147,662	153,333	4.13	4.33	4.18	4.08	4.19	4.18	**3.48**	3.5
10532	234,512	95,469	161,497	206,460	146,022	259,757	90,477	3.27	3.45	3.23	3.23	3.31	3.26	**3.21**	2.9
10093	391,973	15,725	192,337	437,235	156,945	204,355	169,330	**4.13**	7.74	4.43	4.42	5.13	4.90	4.58	3.55
**Average**	180,165	51,788	116,281	191,692	100,980	160,864	115,810	3.80	4.77	3.87	3.58	4.17	3.91	**3.33**	3.33

Moreover, CryoSegNet performs better than Topaz for all the protein types except EMPIAR ID 10093. The average resolution of CryoSegNet with Select 2D is 3.33 Å, about 7% better than 3.58 Å of Topaz and 14% better than 3.87 Å of crYOLO. Remarkably, for EMPIAR ID 10345, the resolution of the density map reconstructed from CryoSegNet is 2.69 Å, which is much higher than all the other methods. Moreover, the average resolution across all test sets resulting from CryoSegNet picked particles (3.33 Å) is comparable to the average 3.33 Å of the density maps built by their original authors possibly with some manual particle picking, and CryoSegNet has a better resolution than the original ones for three out of five proteins, indicating that it can be applied to the existing cryo-EM micrographs in EMPIAR to generate high-quality density maps.

Comparing the results on all the micrographs of the five protein types (Table 3) and the results on a smaller number of micrographs of the same five protein types (Table 1), the average performance of all three methods on the five protein types is improved, indicating that using more micrographs generally improve the quality of reconstructed density maps as expected. Moreover, applying Select 2D to the density map reconstruction improves the resolution of all the three methods on this dataset, even though Select 2D filters out a substantial number of particles including some true ones picked by each method, indicating that other factors such as the quality and representativeness of picked particles are important. This explains why a single particle picking metric such as recall (sensitivity) does not fully correlate with the resolution of reconstructed density maps. The detailed results of the three methods in all the trials can be found in Supplementary Table S3.

**Figure 3:**
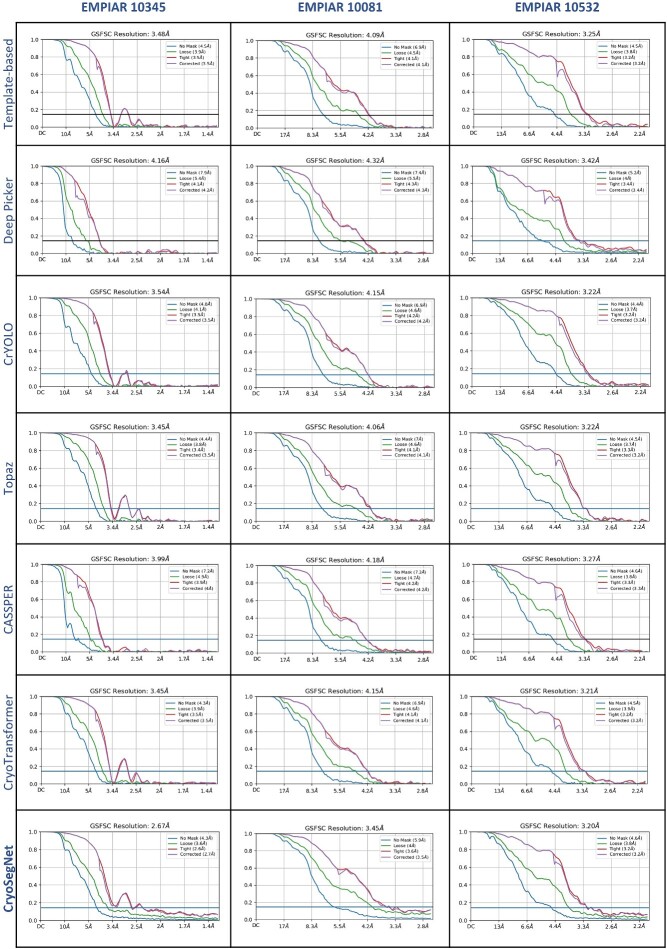
Comparison results for resolution of the 3D density maps of particles picked by the template-based picking, Deep Picker, crYOLO, Topaz, CASSPER, CryoTransformer and CryoSegNet.

**Figure 4:**
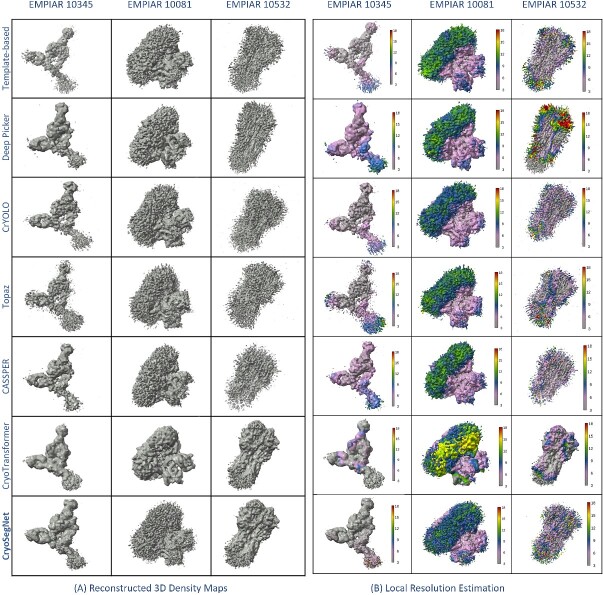
(**A**) Reconstructed 3D density maps and (**B**) local resolution estimation (in Å) of the reconstructed density maps. CryoSegNet has better resolution and local resolution estimation compared to the template-based picking, Deep Picker, CrYOLO, Topaz, CASSPER and CryoTransformer for EMPIAR 10345, EMPIAR 10081 and EMPIAR 10532.

The superiority of CryoSegNet is not only evident in terms of resolution but also in the quality of viewing direction and the representation of various orientations of picked particles. Supplementary Figure S1 showcases the best 2D classes for the five protein types obtained from CryoSegNet, which clearly shows that CryoSegNet picked particles representing many different orientations/views of proteins, which is an important factor of obtaining high-resolution reconstruction of 3D density maps. Further, Fig. 3 illustrates the resolution comparison, Fig. 4 shows the density maps and local resolution estimation of the particles picked by CryoSegNet and the other methods, visually showing that CryoSegNet performs better in four out of five protein types compared to other methods. A detailed illustration of viewing direction comparison, resolution comparison, density maps and local resolution estimation results for all of the protein types is presented in Supplementary Figures S2–S5, respectively.

### Impact of number of micrographs in the resolution of density maps

The resolution of density maps changes with respect to the number of micrographs. For most protein types, the resolution improves. The detailed study is presented in Supplementary Note S2 and Supplementary Table S4.

### Generalization capability of the CryoSegNet

To evaluate the generalization performance of CryoSegNet during testing, we utilized the MMseqs2 tool [41] to calculate the sequence identity between proteins in the training and test datasets. According to the stringent threshold of 25% sequence identity, as utilized by DeepMainmast [42], six (EMPIAR IDs 10081, 10345, 11056, 10532, 10093 and 10017) out of the seven test EMPIAR IDs are dissimilar to the training proteins (less than or equal to 25% sequence identity), while only EMPIAR ID 10028 has 35% sequence identity with some training proteins. On the six dissimilar test proteins (EMPIAR IDs 10081, 10345, 11056, 10532, 10093 and 10017), the average F1-Score of CryoSegNet is 0.74 (see per-protein F1-Score in Table 1), higher than 0.638 of Deep Picker, 0.732 of CrYOLO, 0.718 of Topaz, 0.647 of CASSPER, 0.739 of CryoTransformer and 0.723 of the template-based picking. Further, in terms of the resolution of the 3D density maps reconstructed from the particles picked by the different methods for four dissimilar proteins: EMPIAR IDs 10081, 10345, 10532 and 10093 (see the resolution of the individual proteins in Table 3), the average resolution of CryoSegNet is 4.65 Å, better than 6.58 Å of Deep Picker, 5.13 Å of CrYOLO, 5.06 Å of Topaz, 5.55 Å of CASSPER, 5.27 Å of CryoTransformer and 5.01 Å of the template-based picking, respectively. This demonstrates the CryoSegNet’s capability to generalize effectively over unseen and independent test datasets.

### Enhancing CryoSegNet performance through adaptive weight adjustment (fine-tuning) with predicted labels

In cases where the model performs poorly in predicting protein particles, we can fine-tune the model’s weights by utilizing predicted labels from the pre-trained CryoSegNet and retraining it with a small set of micrographs. We conducted experiments by employing predicted labels from the pre-trained CryoSegNet on 20 sets of micrographs for EMPIAR IDs 11056 and 10017 from the CryoPPP dataset to retrain CryoSegNet starting with the pre-trained weights. The two EMPIAR IDs have sequence identity less than the threshold (25%) with the data used in the training set. For EMPIAR ID 11056, the resolution was improved from 7.17 to 6.13 Å by the fine-tuning, with the number of picked particles increasing from 53,072 to 75,303. Similarly, for EMPIAR ID 10017, the resolution was enhanced from 6.91 to 5.27 Å, with the number of picked particles rising from 10,026 to 33,572. These improved results indicate that the generalization capability of CryoSegNet for new proteins can be further improved by fine-tuning, using the predicted labels. The detailed improved results can be found in Supplementary Table S5.

### Carbon-alpha match score comparison for the 3D structures

We determined the 3D structures from the density maps generated by all methods using the ‘Map to Model’ feature of the Phenix tool [43] and calculated the carbon alpha (Ca) match score [44] by comparing the generated structures with the original ground truth structures. The average Ca match score of the structures built from the CryoSegNet density maps is 19.58%, higher than all the other methods. The detailed results can be found in Supplementary Table S6.

### Training and test time

**Figure 5:**
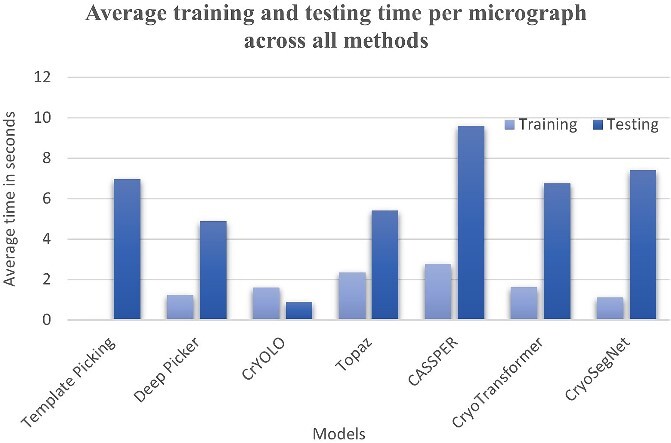
Average training and testing time per micrograph of all methods.

We compared the training and test (inference) time of the AI methods with the same number of CPU cores and GPU (Fig. 5). While CryoSegNet requires less training time than other methods, it needs more time for inference than most other methods due to the incorporation of post-processing steps. However, this increase in the inference time is compensated by its improved accuracy.

### Discussion

Unlike the other AI methods, CryoSegNet employs a symmetric encoder–decoder architecture interconnected by skip connections, optimizing object localization and facilitating effective feature fusion between low-level and high-level features. By eliminating the need for components like non-maximum suppression and anchor generation, CryoSegNet streamlines the particle-picking process, enhancing efficiency. Moreover, the integration of the SAM and a post-processing module further refines particle picking by minimizing false positives. Additionally, its integration techniques such as denoising and attention-gated U-Net, customized loss function along with the SAM model and post-processing, significantly boosts performance compared to their individual application. The ablation study to elucidating the contributions of the different components of CryoSegNet is presented in Supplementary Note S3 and Supplementary Tables S7–S11.

While CryoSegNet demonstrates notable strengths in particle picking, it has weaknesses in picking particles for small proteins, like those in EMPIAR IDs 11056 and 10017. To tackle this limitation, we fine-tuned the CryoSegNet model using predicted labels from a pre-trained model, which significantly enhanced particle picking for these proteins (see two examples in section Enhancing CryoSegNet performance through adaptive weight adjustment (fine-tuning) with predicted labels). These findings underscore the effectiveness of augmenting the training dataset with predicted labels micrographs of small proteins, as detailed in Supplementary Table S5. Further strengths and weaknesses of different metrics used in evaluation of particle picking and limitations of CryoSegNet are discussed in Supplementary Note S4.

## Materials and methods

### Dataset

We employed an extensive and diverse dataset (CryoPPP) to train, validate and test CryoSegNet. Specifically, we utilized the micrographs of 22 EMPIAR IDs (protein types) from the CryoPPP for training and validation. We allocated 80% of the micrographs from each of the 22 protein types for training and the remaining 20% for validation. The training dataset consisted of 4,948 micrographs, while our validation set was comprised of 1,244 micrographs. The details of the training and validation datasets are presented in Supplementary Table S12. For the independent test, we selected a separate set of seven different EMPIAR IDs from the CryoPPP dataset. The details of the dataset are described in Supplementary Table S13.

### Prediction methods

#### Attention-gated U-Net

**Figure 6:**
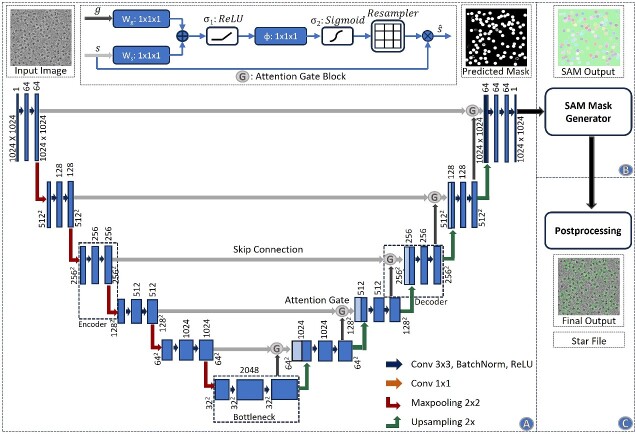
Architecture of the CryoSegNet model. (**A**) The attention-gated U-Net to predict segmentation mask for a micrograph. The numbers in the top of the rectangular slices indicate the number of channels and in the bottom indicate the size of the output. The U-Net has five encoders, one bottleneck component and five decoders. The skip connection from each encoder to its corresponding decoder goes through an attention gated block. Each attention block for a decoder also takes an input from its previous decoder or the bottleneck component. The details of the attention block are illustrated at the middle top. (**B**) The SAM mask generator takes input from the output of the U-Net model and outputs bounding box coordinates and intersection over union score for each predicted protein particle in the micrograph. (**C**) The postprocessing module outputs the star file containing picked particles and processed output micrographs based on the thresholding criterion for each protein type.

The advent of deep learning architectures like U-Net has greatly simplified segmentation tasks in biomedical images like localizing mitochondria cells and brain tumors. In this work, we designed a special U-Net architecture (Fig. 6) for cryo-EM protein particle picking by making it deeper and introducing an attention mechanism into it, considering the large size of the cryo-EM micrographs and the nature of protein particles in the micrographs. Cryo-EM micrographs often contain objects that are not actual single protein particles, such as ice patches, protein aggregates and false particles along the carbon edges. These false positives can negatively degrade the resolution of the final 3D structures reconstructed from the particles. Therefore, it is important to prioritize the picking of true protein particles for an accurate segmentation. Thus, we added attention gates in the expanding path of the U-Net architecture to put a significant emphasis on true protein particles. Our model consists of five encoder blocks in the contracting path, a bottleneck layer and five decoder blocks in the expanding path, each equipped with attention gates. This architecture modification can effectively handle the complexity of cryo-EM micrographs and achieve the precise segmentation of protein particles.

The U-Net takes a cryo-EM micrograph of size 1024 × 1024 as input and outputs a segmentation mask of size 1024 × 1024. A loss function that combines both binary cross entropy loss and dice loss [33] is used to measure prediction error in training. The former allows for measuring individual pixel error independently, while the latter assesses the degree of dissimilarity between the predicted segmentation mask and the ground truth segmentation masks. By minimizing these two, the network is trained to achieve more accurate segmentation of protein particles. The output of the U-Net is used as input for SAM’s automatic mask generator for further segmentation.

#### SAM automatic mask generator

Meta’s SAM has achieved great success in segmenting objects in many images. However, directly applying the pretrained SAM to cryo-EM micrographs can only pick very few particles because cryo-EM images are very different from the images used to train SAM. Fine-tuning (retraining) the SAM’s mask decoder on cryo-EM micrographs for thousands of epochs improved results over the original SAM but still could not achieved satisfactory results and performed worse than the state-of-the-art deep learning particle pickers such as Topaz. After many trials, we finally devised a hybrid approach that combines the U-Net model with SAM’s automatic mask generator, which is proved to be highly effective for particle picking.

In the hybrid approach, the output of the attention-gated U-Net is fed to the SAM’s automatic mask generator module. This module was tailored for automatic mask generation for input images and was trained on the SA-1B dataset. Firstly, it generates the masks from a grid of points, incorporating various scales of the original and zoomed images. Then, cropping is performed using a regular grid of points, and any masks intersecting crop boundaries are discarded. Redundant masks are then eliminated through non-maximum suppression with an intersection over union (IoU) threshold of 0.7, retaining only masks with confidence scores exceeding 88.0. Subsequent processing steps refine the masks by removing small artifacts and filling minor gaps, which are particularly important considering the high-noise and low-contrast characteristics of cryo-EM micrographs.

These refined masks as well as the IoU scores and bounding box coordinates for each picked protein particle within the micrographs are then passed through our postprocessing modules below designed to filter out some false positives and improve the precision of particle picking.

#### Postprocessing

The output generated by SAM’s automatic mask generator undergoes the additional postprocessing to generate .star files, which contain coordinate information for protein particles. Supplementary Algorithm S1 outlines the complete steps of the postprocessing.

### Data preprocessing

#### Denoising of micrographs

The cryo-EM micrographs have low contrast and low SNR, necessitating the use of image denoising techniques before using them as input for the U-Net. Supplementary Figure S6 illustrates the denoising techniques used for preprocessing cryo-EM micrographs. The image preprocessing pipeline begins with reading the images in the mrc format and applying a Gaussian filter. Subsequently, the images are standard normalized and converted to grayscale, with pixel values ranging from 0 to 255. To effectively reduce noise while preserving image details, the Fast Non-Local Means (FastNLMeans) denoising technique [23, 31] is applied, followed by noise mitigation through Weiner filtering [23, 32].

To enhance the contrast of cryo-EM micrographs and improve the visibility of protein particles, the contrast limited adaptive histogram equalization (CLAHE) technique is then incorporated. The CLAHE technique is widely used to enhance images with regions of non-uniform illumination and low contrast. Finally, the CLAHE-equalized image is used as a guided image to the Weiner-filtered image to perform guided filtering, allowing selective smoothing and enhancement of the cryo-EM micrographs while preserving edges and fine details.

#### Standardization of inputs and labels

The CryoPPP dataset comprises diverse protein types, each with varying micrograph sizes. Image size ranges from as low as (3710, 3710) to as high as (7676, 7420). For the uniformity in the training process, we resized all the micrographs to (1024, 1024) after denoising them and before feeding them to the U-Net model. From the ground truth coordinate files in the .csv format, containing information like centers of the particles and corresponding diameters, we created a separate ground-truth segmentation mask for each micrograph. This mask was then resized to (1024, 1024). The input micrograph was fed to the network for training, while the ground-truth segmentation mask was utilized as a target and compared with the output segmentation mask for calculation of loss. Supplementary Figure S7 shows a sample denoised image and its corresponding ground-truth segmentation mask.

### Training

The attention-gated U-Net of CryoSegNet was trained using denoised and resized micrographs of 22 different EMPIAR IDs from CryoPPP dataset. The training was done with a batch size of 6, learning rate of 0.0001 for 200 epochs with a combined loss function of the dice loss and binary cross entropy on NVIDIA A100 80GB GPU.

**Key Points**

- A deep learning method (CryoSegNet) integrating an attention-gated U-Net and the foundational Segment Anything Model was developed to pick protein particles in cryo-EM images.
- CryoSegNet has both high precision and recall for picking protein particles.
- The average resolution of cryo-EM density maps built from CryoSegNet picked particles on a dataset is 7–14% better than two widely used deep learning methods for particle picking.
- CryoSegNet can be used to automate the laborious cryo-EM particle picking process and improve the quality of the cryo-EM density maps built from cryo-EM image data.

## Supplementary Material

Supplementary_Information_Briefings_in_Bioinformatics_bbae282

## Data Availability

The dataset for this study is available on https://github.com/BioinfoMachineLearning/cryoppp and https://zenodo.org/record/7934683. The source code is available on https://github.com/jianlin-cheng/CryoSegNet.

## Author Biographies

**Rajan Gyawali** is currently pursuing a PhD degree in computer science at University of Missouri, Columbia, MO, USA. His research interests include bioinformatics, biomedical imaging and protein structure determination.

**Ashwin Dhakal** is currently pursuing a PhD degree in computer science at University of Missouri, Columbia, MO, USA. His research interests include bioinformatics and artificial intelligence.

**Liguo Wang** is a director of scientific operations at Laboratory for BioMolecular Structure, Brookhaven National Laboratory, Upton, NY, USA. His research focuses on cryo-EM electron microscopy.

**Jianlin Cheng** is a professor and director of Bioinformatics and Machine Learning Lab at University of Missouri, Columbia, MO, USA. His research focuses on bioinformatics, machine learning and artificial intelligence.
