# Supplementary material for: CryoSegNet: accurate cryo-EM protein particle picking by integrating the foundational AI image segmentation model and attention-gated U-Net
Source: Brief Bioinform. 2024 Jun 11;25(4):bbae282. doi: 10.1093/bib/bbae282 (PMC11165428; doi:10.1093/bib/bbae282)
Supplement: Supplementary_Information_Briefings_in_Bioinformatics_bbae282 [file supplementary_information_briefings_in_bioinformatics_bbae282.docx]

*Briefings in Bioinformatics**
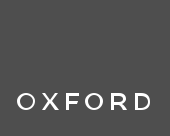
*

**CryoSegNet: Accurate cryo-EM protein particle picking by integrating the foundational AI image segmentation model and specialized U-Net**

Rajan Gyawali[
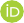
](https://orcid.org/0000-0002-7052-4964),^1,^ ^2^ Ashwin Dhakal[
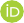
](https://orcid.org/0000-0002-4047-9947),^1,^ ^2^ Liguo Wang^3^ and Jianlin Cheng[
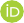
](https://orcid.org/0000-0003-0305-2853)^1,^ ^2,^ *

^1^Department of Electrical Engineering and Computer Science, University of Missouri, Columbia, 65211, MO, USA

^2^NextGen Precision Health, University of Missouri, Columbia, 65211, MO, USA

^3^Laboratory for BioMolecular Structure (LBMS), Brookhaven National Laboratory, Upton, 11973, NY, USA

*Corresponding author: Jianlin Cheng ([chengji@missouri.edu](mailto:chengji@missouri.edu))

**This supplementary information contains Supplementary Notes S1 - S4, Supplementary Figures S1 - S9, Supplementary Tables S1 - S14, and Supplementary Algorithm S1.**

**Supplementary Notes**

***Supplementary Note S1:*** ***Parameters in CrYOLO and Topaz***

There are few parameters in CrYOLO [1] and Topaz [2] that affect the quality and number of picked protein particles. While using CrYOLO, two parameters can significantly impact the results. One such parameter is the confidence threshold, which has a default value of 0.3. Increasing this parameter leads to a lower number of picked particles, while a value less than 0.3 tends to result in a larger number of picked particles. We tested various threshold values for each EMPIAR ID in the test dataset [3], and have utilized the default threshold value that yielded the best results. Another parameter influencing particle picking in CrYOLO is the network architecture it offers. Among the three different architectures, namely “YoLO”, “CrYOLO” and “PhosaurusNet”, we opted for PhosaurusNet, as it can effectively pick small particles and eliminate picking on the carbon edge.

In the case of Topaz, there are also a few parameters that can affect the results of particle picking. One major parameter is the architecture, and we utilized the ResNet16 architecture for particle picking. Another critical parameter is the "radius of extracted regions," which significantly influences the number of picked particles. The default value of 7 often leads to overlapping and duplicates. For instance, in EMPIAR ID 10028 [4] using default value of 7 leads to 169,733 particles. Increasing this value to 10, provides 88,771 particles reducing the duplicates/overlapping particles in high number. Further, increasing the threshold value to 20 leads to very minimal duplicates/overlapping particles yielding only 52,588 particles. For EMPIAR ID 10093 [5] and EMPIAR ID 10017 [6] we used the radius of extracted regions as 15 and for rest of the EMPIAR IDs value of 12 yielded the minimum number of duplicates/overlapping particles. This parameter selection depends from person to person, but the basic idea is to increase the radius parameter by some value and observe if there are any duplicates. The best value can be determined by finding the size and total structure weight of the protein.

***Supplementary Note S2:*** ***Impact of number of micrographs in the resolution of density maps***

We further analyzed the impact of the number of micrographs on the resolution of the reconstructed 3D density maps for the five protein types by comparing the performance of CryoSegNet on a few hundred micrographs in CryoPPP test dataset and the full set of micrographs in EMPIAR (**Supplementary Table S4**). The results show that augmenting the number of micrographs generally results in an increased number of protein particles at different viewing directions on four of five protein types, thereby contributing to improved resolution. However, if the number of particles with different conformations remains unchanged, increasing the number of micrographs does not significantly impact the final 3D resolution. For example, EMPIAR ID 10028 (ribosome), the resolution of using 300 micrographs is 2.72 Å, which is the same as that of using 600 micrographs.

***Supplementary Note S3:*** ***An Ablation Study of CryoSegNet and the Foundational AI Model (SAM)***

We performed a series of experiments to compare CryoSegNet and different ways of using the foundational AI model (i.e., SAM [9]) for protein particle picking from cryo-EM micrographs. We first explored directly applying the pretrained SAM in its original form to the cryo-EM micrographs, which yielded very unsatisfactory results due to the inherent new challenges posed by cryo-EM micrographs (very low contrast and a low SNR) not seen in the data used to trained SAM. Only a few protein particles with distinct contrast and high SNR can usually be segmented by SAM. To address this limitation, we then fine-tuned the SAM’s mask decoder by training it on our dataset for 2000 epochs. We conducted this training by using the weights of three versions of SAM [9](i.e., ViT-H, ViT-L and ViT-B) as start point, respectively. The three fined tuned SAM generally performed better than the original SAM. Notably, the best segmentation results of the three were achieved by fine-tuning the ViT-H model. Some of the results of segmentation by fine-tuned SAM are shown in **Supplementary Figure S8**. Finally, we explored the approach used by CryoSegNet combining the U-Net model with the SAM’s automatic mask generator, which was performed by feeding the output of the former into the latter to generate the segmentation results. All the three approaches above were applied to the same micrographs denoised by the image processing techniques that enhanced the effectiveness of all the approaches. **Supplementary Figure S9** illustrates the particle segmentation results of the three approaches on one typical example (EMPIAR ID 10028), which clearly demonstrate that combining the U-Net with SAM performs much better than the fine-tuned SAM that in turn substantially outperforms the original SAM. The results show that the U-Net is able to convert original cryo-EM micrographs not well understood by SAM to the segmentation maps that can be handled by it well to improve its performance for particle picking.

Additionally, before finalizing the U-Net architecture, we thoroughly experimented with various components and hyperparameters to assess their impact. We investigated the influence of using denoised micrographs versus non-denoised ones, observing enhanced training performance with denoised micrographs, as demonstrated in **Supplementary Table S7**. Exploring different configurations of encoder and decoder blocks, ranging from 4 to 6, we determined that optimal performance was achieved with 5 blocks, as evidenced in **Supplementary Table S8**. Furthermore, we examined the effect of employing different loss functions and discovered that combining binary cross-entropy with Dice loss yielded superior performance compared to using them individually, as shown in **Supplementary Table S9**. Additionally, incorporating attention gates before the decoder block resulted in improved segmentation results, as illustrated in **Supplementary Table S10**. Moreover, by experimenting with varying the size of the input micrograph, ranging from 512 x 512 to 2048 x 2048 pixels, we concluded that the best results were achieved with an input size of 1024 x 1024 pixels, as detailed in **Supplementary Table S11**.

During the comparison with Topaz in terms of the resolution of reconstructed density maps, we explored the various versions of the Topaz architecture to get its best results. It was observed that the results were superior when using ResNet 16 (64 units) in comparison to ResNet 8 (32 units). Additionally, we conducted the experiments by adjusting the particle threshold parameter, ranging from default value 0 to 2 with an increment of 1. These results are presented in **Supplementary Table S*14***. A higher threshold can reduce the number of duplicate and overlapped particles but may also reduce the recall of particle the picking. The results show that increasing the threshold does not improve the resolution of the reconstructed density maps. Therefore, the best result of Topaz achieved at the default threshold of 0 is used to compare with CryoSegNet.

***Supplementary Note S4*: *The strength and weaknesses of different metrics of evaluating particle picking and the limitation of CryoSegNet***

As cryo-EM particle picking is still a young field, the metrics of evaluating its performance have not been well established. In this work, we use the standard image classification metrics including precision, recall, F1-score and Dice score as well as the specialized evaluation metrics such as the resolution of density maps reconstructed from picked particles that users care about most. Each classification metric is an indicator of the performance of the particle picking but none of them is 100% correlated with the resolution of density maps. The correlation between each of the classification metrics (F1-score, precision, Dice score, and recall) and the resolution value (quality) of the density maps reconstructed from CryoSegNet with Select 2D is -0.88, -0.94, -0.91, and -0.78. The correlation is computed from the classification metric values for five protein types (see **Table 1** in the main manuscript) and the resolution values of the density maps (see **Table 3** in the main manuscript). The correlation shows that the F1-score, precision and Dice score are the rather informative classification metric for predicting the quality of reconstructed density maps, which have a much stronger correlation with the resolution of the reconstructed density maps than recall. The recall is the least informative probably because when there are enough picked particles, the quality or the representativeness of the particles may be more important and low-quality or false particles may severely reduce the quality of the reconstructed density maps. Moreover, none of the standard classification metric can perfectly predict the resolution of the reconstructed density maps because the density reconstruction process is very complicated, and its outcome depends on many factors such as the quality and diversity of true particles picked that the standard classification metrics cannot measure. Therefore, the resolution of the reconstructed cryo-EM density maps is the most important metric of assessing the effectiveness of a particle picking method.

CryoSegNet offers a more reliable and automated solution for cryo-EM particle picking compared to conventional manual and template-based methods. It eliminates labor-intensive manual selection, reducing human bias and increasing objectivity. The density maps reconstructed from CryoSegNet-picked particles exhibit higher average resolution than those from manually picked particles, suggesting its potential to replace time-consuming manual or template-based methods. Compared to automated machine learning methods crYOLO and Topaz, CryoSegNet significantly improves map resolution and outperforms them in terms of F1-score, precision, and Dice score, metrics strongly correlated with map resolution. Hence, CryoSegNet shows promise for more accurate picking from existing or new cryo-EM data previously processed by other methods.

There are still some challenges faced by AI-based particle picking methods including CryoSegNet on some datasets like EMPIAR ID 10532 and EMPIAR ID 10093 that have few samples representing rare protein view orientations, some of which could be missed by the automated AI methods. In the two cases, they performed worse than the blob-based picking in RELION used by the original authors (see **Table 3** in the main manuscript). One reason is that the blob-based picking was used by the authors to capture rare but diverse protein-like objects, even though it might also pick undesired false particles that required subsequent steps of false positive removal. Another limitation is the requirement of high computing resources for training CryoSegNet on large cryo-EM datasets. We will explore better optimization techniques to address this issue in the future.

**Supplementary Figures**


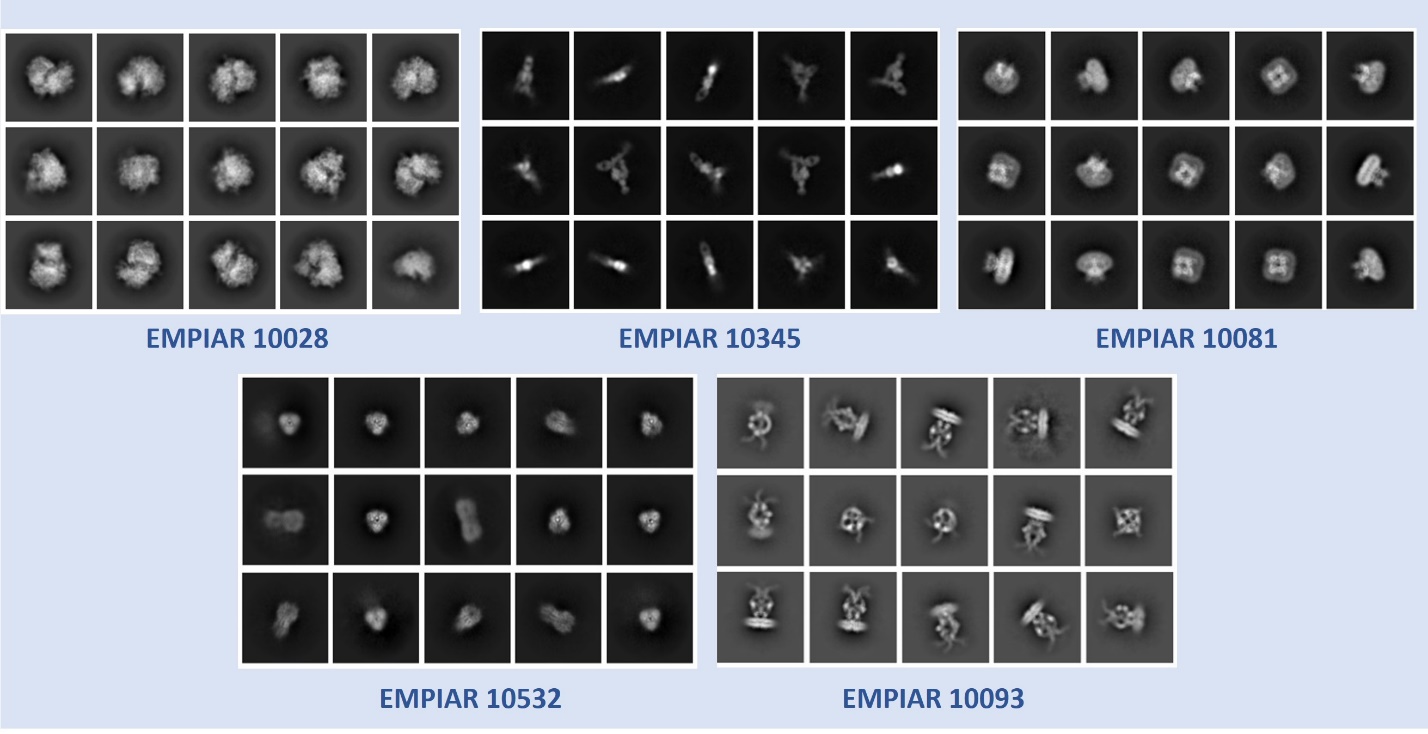


**Supplementary Figure S1****:** 2D classes from particles picked by CryoSegNet for EMPIAR 10081, EMPIAR 10345, EMPIAR 10532, EMPIAR 10028 and EMPIAR 10093. These classes show particles with multiple orientations that have been picked by CryoSegNet.


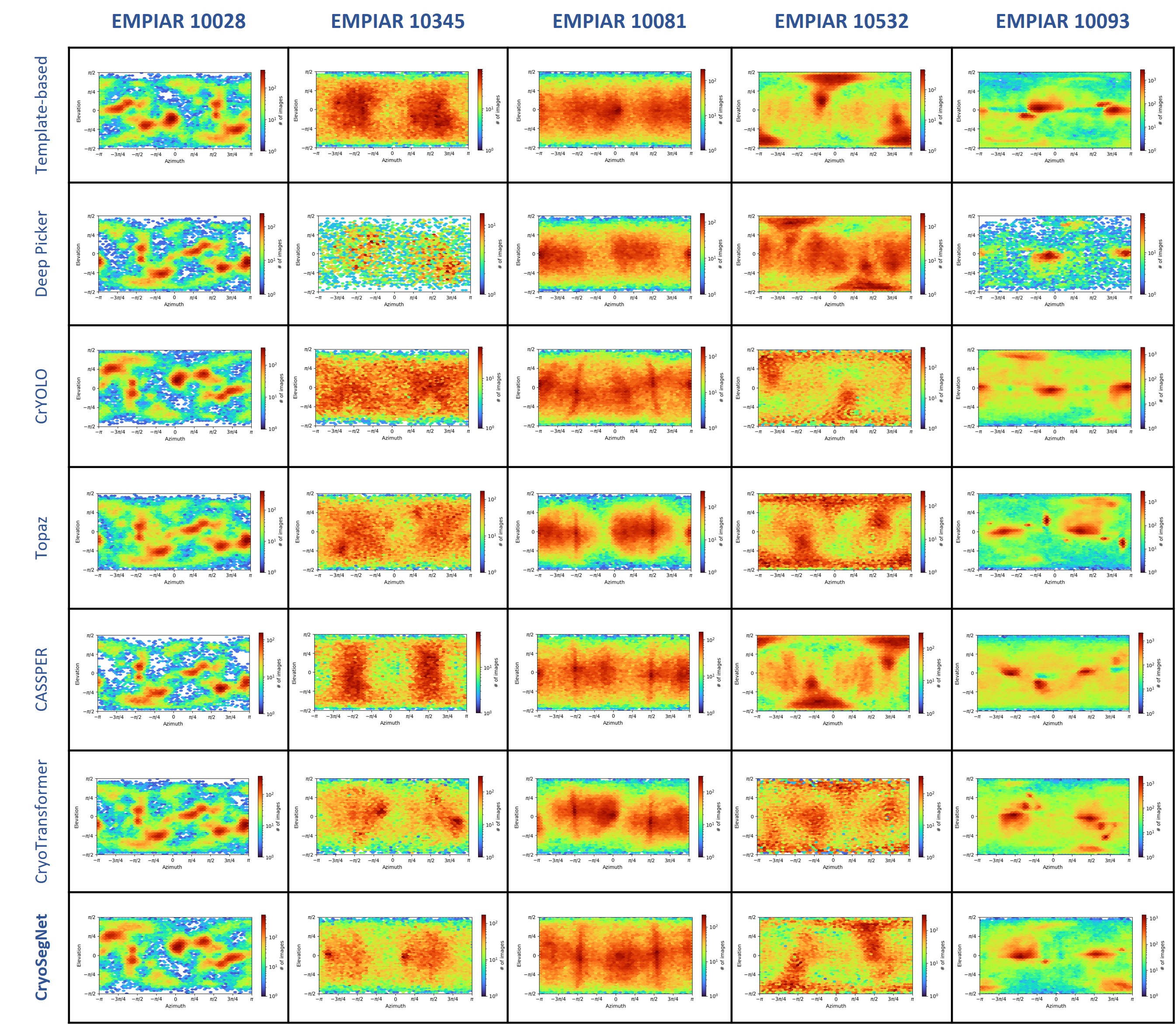


**Supplementary Figure S2:** Comparison results for viewing direction of particles picked by template-based picking, Deep Picker, crYOLO, Topaz, CASSPER, CryoTransformer and CryoSegNet. From the viewing direction plots, it is observed that Deep Picker, crYOLO and CASSPER picks very few particles and misses many true protein particles while CryoSegNet picks particles with multiple orientations/views.


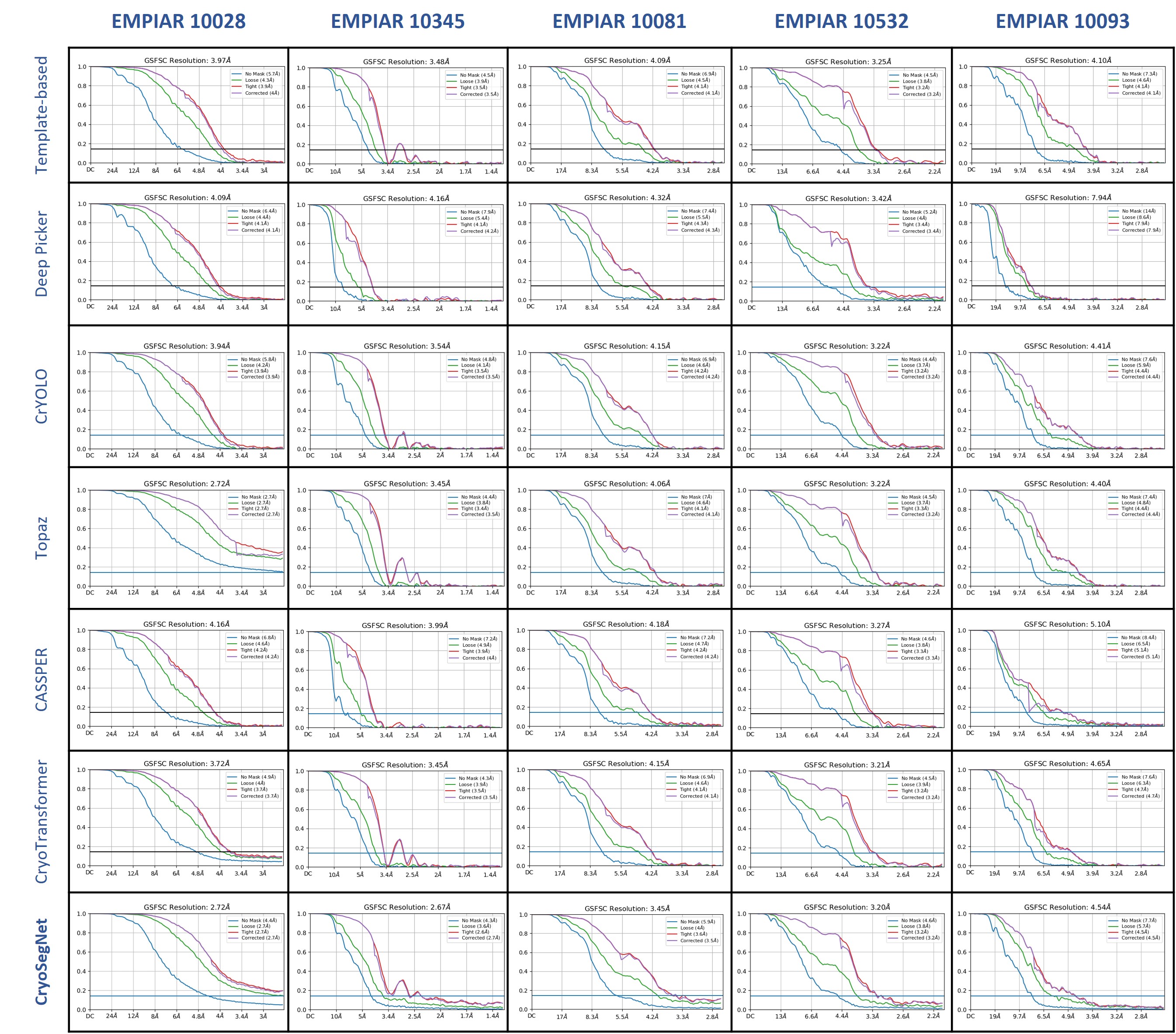


**Supplementary Figure S3:** Comparison results for resolution of density maps of particles picked by template-based picking, Deep Picker, crYOLO, Topaz, CASSPER, CryoTransformer and CryoSegNet. CryoSegNet has better resolution than other methods in most protein types.


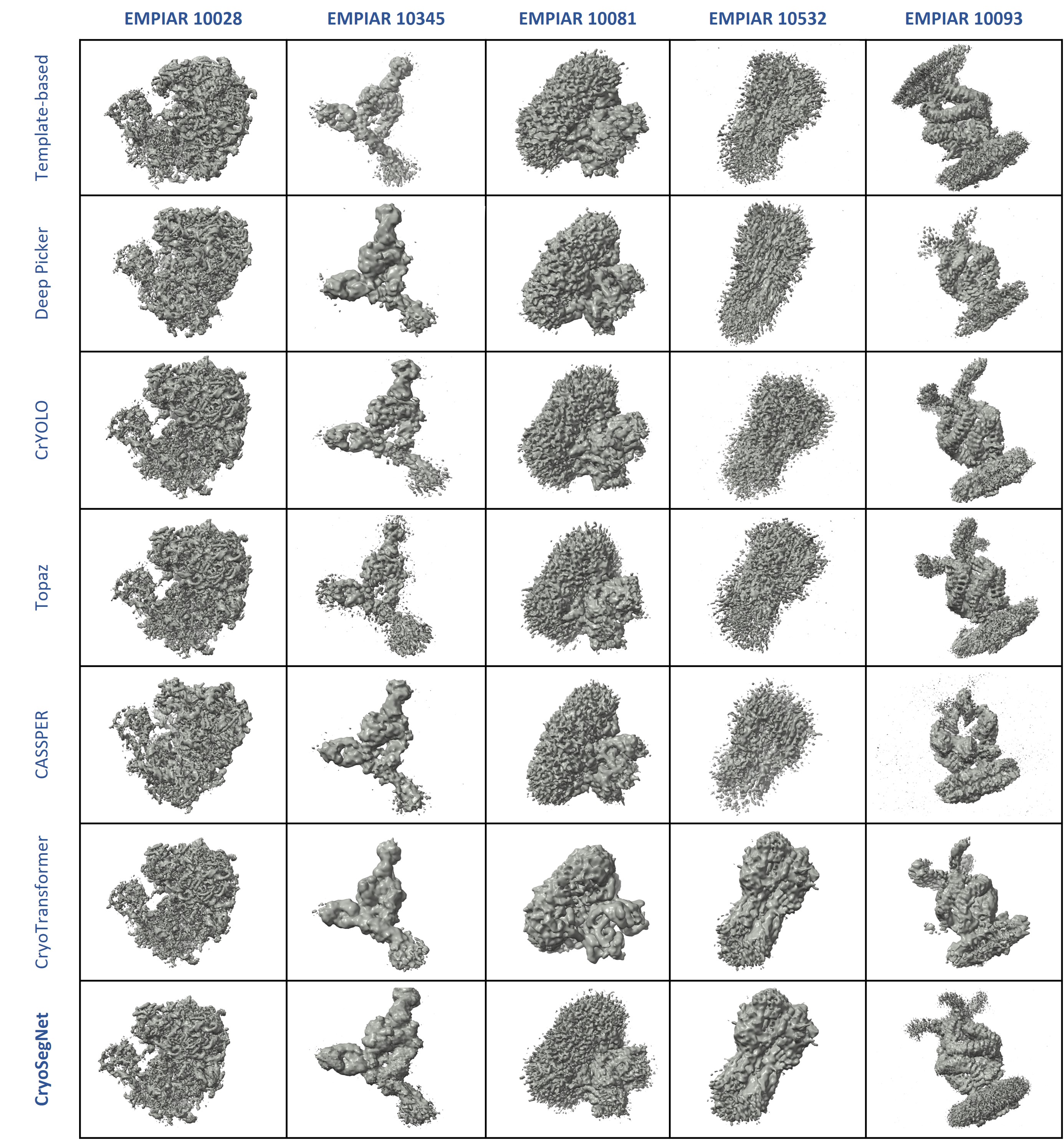


**Supplementary Figure S4:** Comparison results for density maps of particles picked by template-based picking, Deep Picker, crYOLO, Topaz, CASSPER, CryoTransformer and CryoSegNet. CryoSegNet outputs high resolution density maps compared to other methods in most protein types.


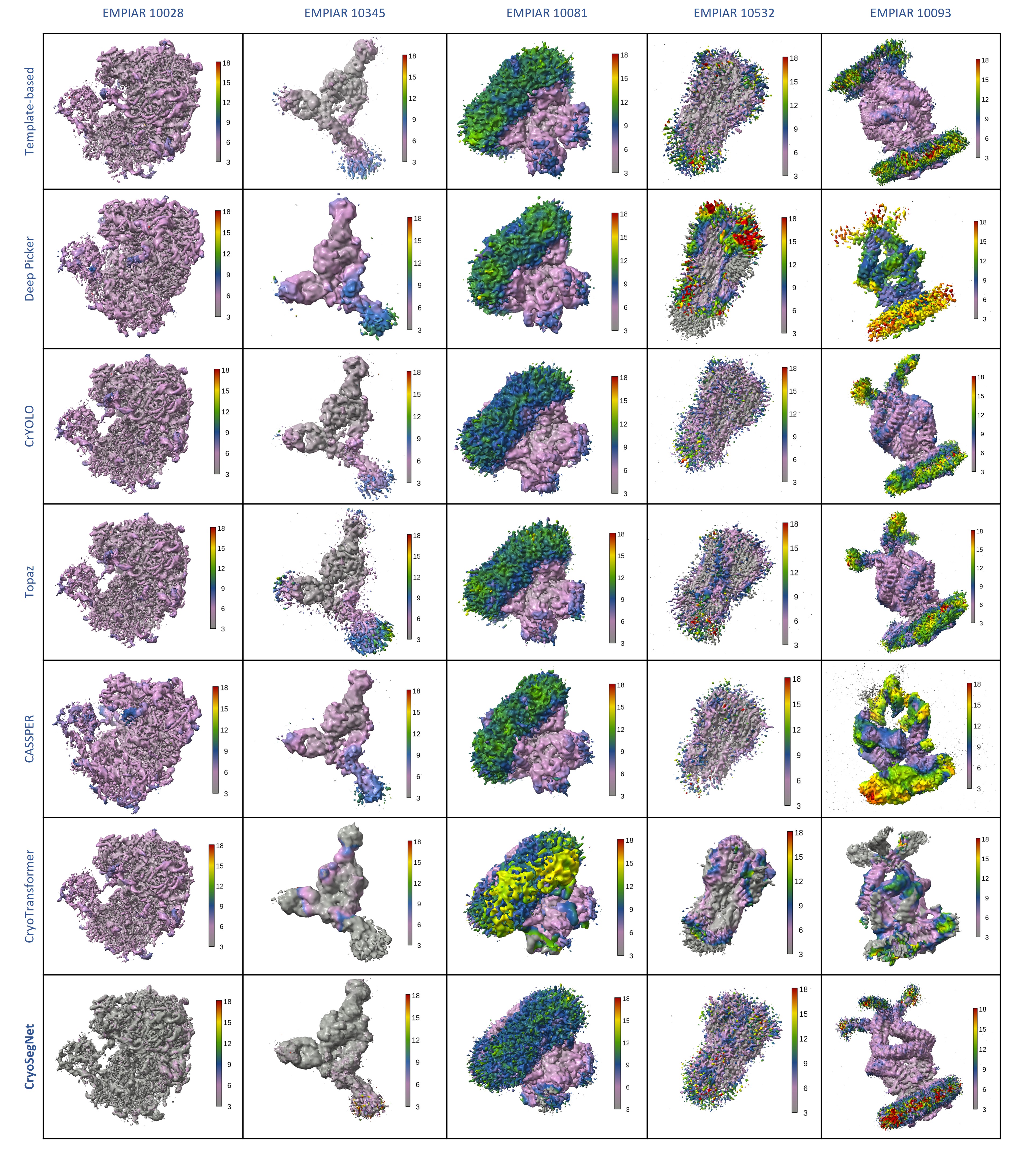


**Supplementary Figure S5:** Comparison results for local resolution estimation of density maps of particles picked by template-based picking, Deep Picker, crYOLO, Topaz, CASSPER, CryoTransformer and CryoSegNet. The color scale (in Angstrom) displayed in the right of the map represents high-resolution areas in gray and low-resolution regions in red.


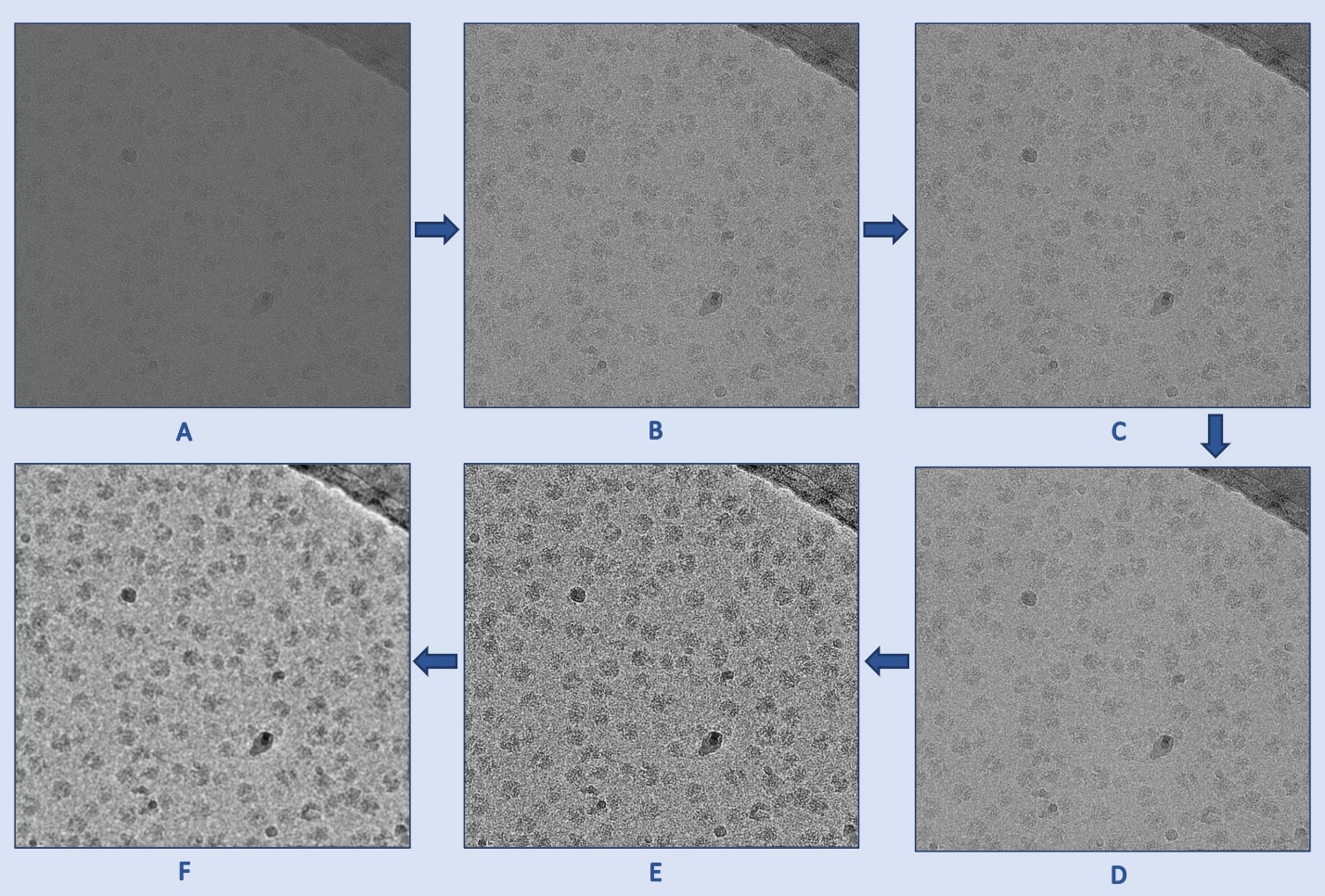


**Supplementary Figure S6:** The denoising process used to preprocess cryo-EM micrographs. **(A)** An original low contrast and low SNR cryo-EM micrograph (EMPIAR ID 10406). **(B)** A standard normalized cryo-EM image. **(C)** A denoised image using FastNLMeans technique. **(D)** Weiner filter applied to the (C) for further denoising. **(E)** Contrast enhancement using CLAHE technique. **(F)** Guided filtered image with (E) as a guided image to the Weiner filtered image (D). As shown in these images, the preprocessing techniques gradually improve the contrast and SNR of the micrograph.


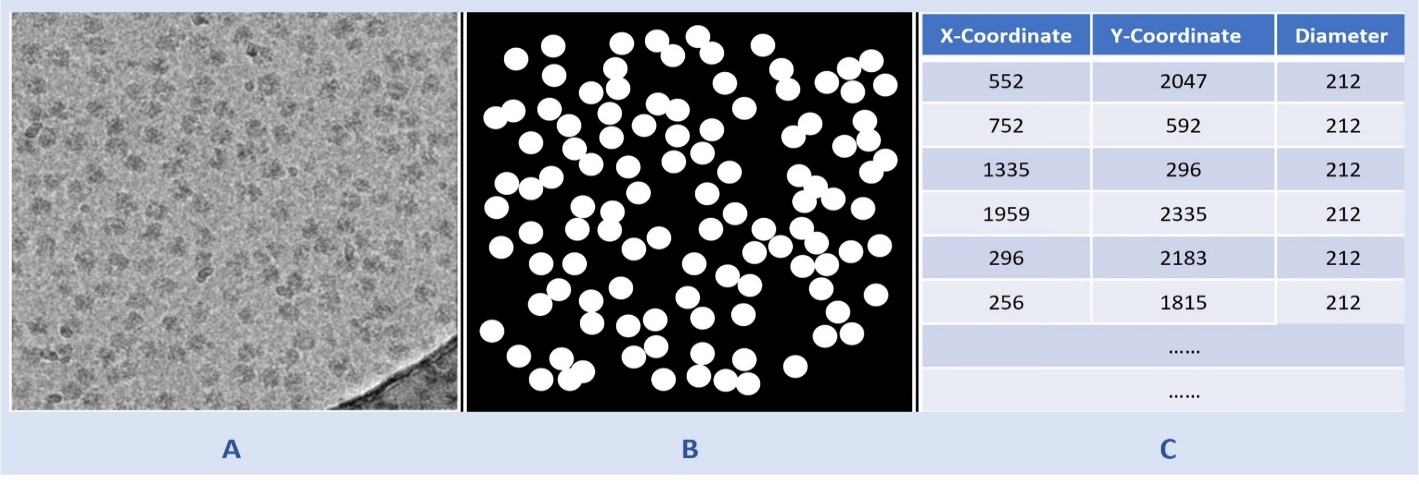


**Supplementary Figure S7:** Illustration of data preparation for training the U-Net model. **(A)** A denoised cryo-EM micrograph (EMPIAR ID 10406) as input. **(B)** The ground truth segmentation mask. **(C)** The information from the ground truth coordinate file with x-coordinate and y-coordinate of center of protein particles and corresponding diameters used to generate (B).


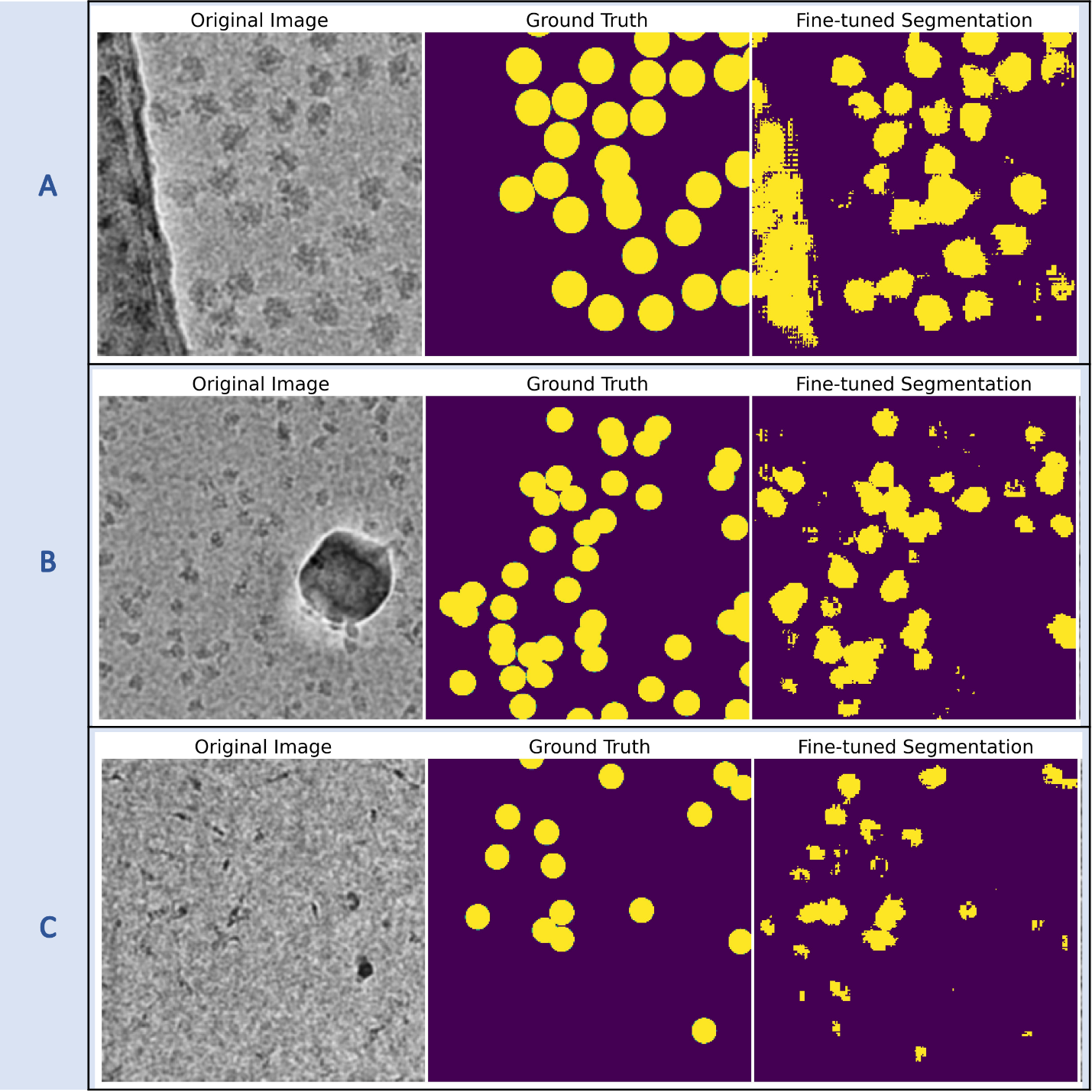


**Supplementary Figure S8**: Segmentation results of fine-tuned SAM **(A)**The whole carbon region is segmented (EMPIAR ID 10532) **(B)** Protein particles of certain orientations are only segmented (EMPIAR ID 10081) **(C)** Some true protein particles are missed and more false positives are segmented (EMPIAR ID 10345)


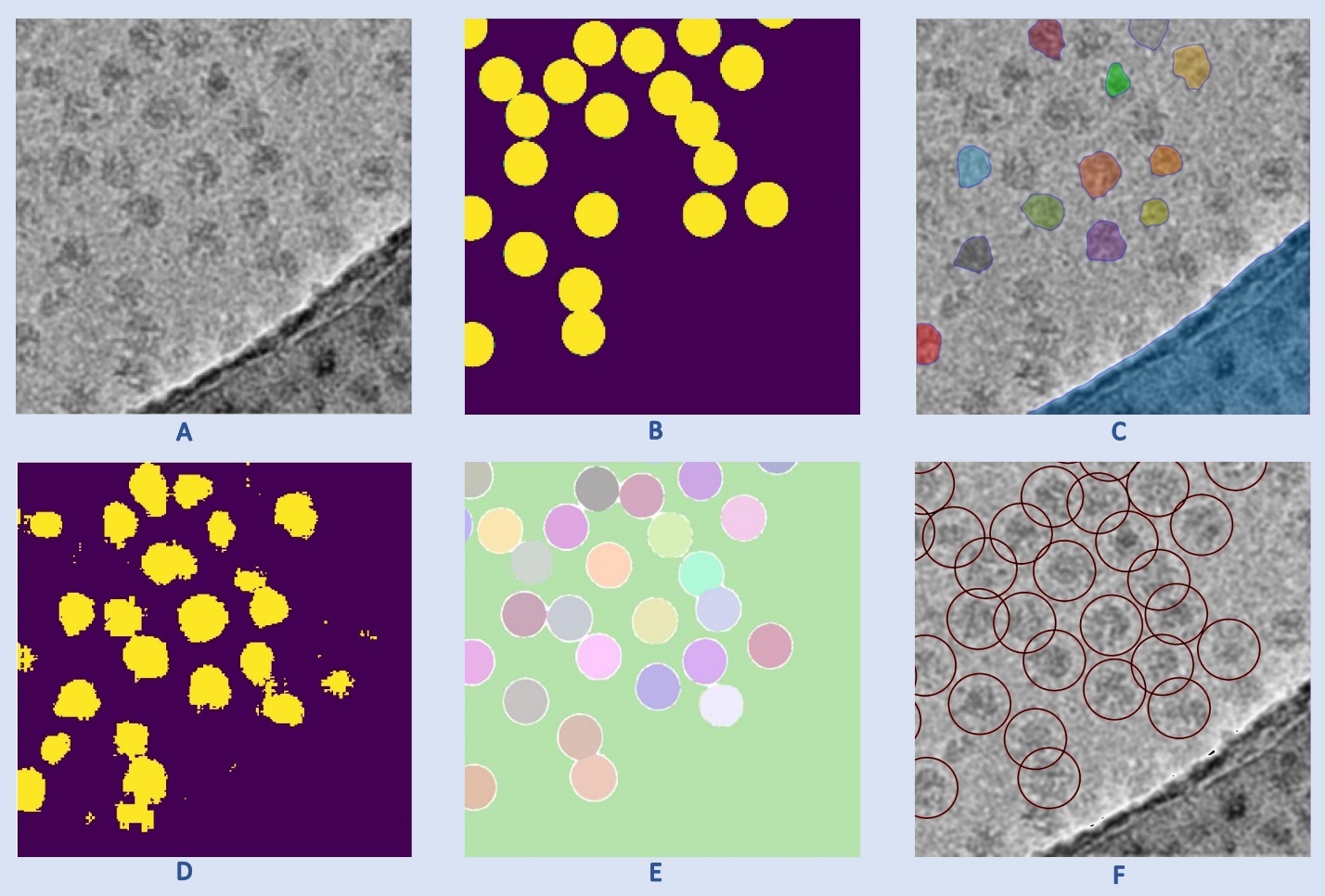


**Supplementary Figure S9:** Results of applying different approaches of using SAM for particle picking. **(A)** Input cryo-EM micrograph (a small patch of a full micrograph from EMPIAR ID 10028). **(B)** Ground truth mask for (A). **(C)** Segmentation result for SAM in its original form. **(D)** Segmentation result for the fine-tuned SAM. **(E)** Segmentation result for SAM used with the U-Net in CryoSegNet. **(F)** Final result for CryoSegNet.

**Supplementary Tables**

**Supplementary Table S1:** Comparison of precision, recall, F1-score and Dice score for different modules of CryoSegNet

| **EMPIAR ID** | **Evaluation metrics from output of UNET module** | | | | **Evaluation metrics from output of SAM module** | | | | **Evaluation metrics from Postprocessing module** | | | |
| --- | --- | --- | --- | --- | --- | --- | --- | --- | --- | --- | --- | --- |
|  | **Precision** | **Recall** | **F1 Score** | **Dice Score** | **Precision** | **Recall** | **F1 Score** | **Dice Score** | **Precision** | **Recall** | **F1 Score** | **Dice Score** |
| 10028 | 0.862 | 0.828 | 0.844 | 0.819 | 0.729 | 0.955 | 0.826 | 0.810 | 0.833 | 0.944 | 0.885 | 0.859 |
| 10081 | 0.847 | 0.959 | 0.899 | 0.866 | 0.783 | 0.943 | 0.856 | 0.841 | 0.835 | 0.922 | 0.876 | 0.876 |
| 10345 | 0.569 | 0.872 | 0.688 | 0.610 | 0.656 | 0.911 | 0.763 | 0.725 | 0.746 | 0.920 | 0.824 | 0.743 |
| 11056 | 0.793 | 0.592 | 0.678 | 0.629 | 0.730 | 0.770 | 0.749 | 0.686 | 0.757 | 0.687 | 0.720 | 0.663 |
| 10532 | 0.860 | 0.507 | 0.638 | 0.509 | 0.757 | 0.768 | 0.763 | 0.726 | 0.796 | 0.628 | 0.702 | 0.649 |
| 10093 | 0.454 | 0.944 | 0.613 | 0.570 | 0.607 | 0.661 | 0.633 | 0.592 | 0.716 | 0.515 | 0.600 | 0.537 |
| 10017 | 0.847 | 0.473 | 0.608 | 0.558 | 0.840 | 0.732 | 0.783 | 0.754 | 0.859 | 0.616 | 0.718 | 0.703 |
| Average | 0.747 | 0.739 | 0.710 | 0.652 | 0.729 | 0.820 | 0.768 | 0.733 | 0.792 | 0.747 | 0.761 | 0.719 |

**Supplementary Table S2**: Comparison of 3D resolution of CryoSegNet on test dataset with template-based picking, Deep Picker, crYOLO, Topaz, CASSPER and CryoTransformer. Bold font denotes the best resolution across 3 trials and bold underlined font represents the best average resolution across all methods.

| **EMPIAR ID** | **Number of Micrographs** | **Method** | **Without Select 2D** | | | | | **With Select 2D** | | | | |
| --- | --- | --- | --- | --- | --- | --- | --- | --- | --- | --- | --- | --- |
|  |  |  | **Resolution for 3 Trials (Å)** | | | **Average Resolution (Å)** | **Number of Particles** | **Resolution for 3 Trials (Å)** | | | **Average Resolution (Å)** | **Number of Particles** |
|  |  |  | **1** | **2** | **3** |  |  | **1** | **2** | **3** |  |  |
| 10028 | 300 | *Template-based* | 4.14 | 4.15 | **4.11** | 4.13 | 35,025 | 4.12 | **4.11** | 4.12 | 4.12 | 32,183 |
|  |  | *Deep Picker* | 4.11 | **4.10** | 4.11 | 4.11 | 32,334 | 4.11 | 4.09 | **4.08** | 4.09 | 30,242 |
|  |  | *CrYOLO* | **4.13** | 4.14 | 4.13 | 4.13 | 32,687 | 4.12 | **4.11** | 4.11 | 4.11 | 31,699 |
|  |  | *Topaz* | 4.01 | 4.05 | **3.98** | 4.01 | 52,588 | **3.93** | 4.02 | 3.96 | 3.97 | 35,514 |
|  |  | *CASSPER* | 4.47 | 4.48 | **4.51** | 4.49 | 16,504 | 4.43 | 4.44 | **4.42** | 4.43 | 15,637 |
|  |  | *CryoTransformer* | 4.17 | 4.16 | **4.16** | 4.16 | 49,495 | **3.82** | 3.91 | 3.86 | 3.86 | 40,488 |
|  |  | *CryoSegNet* | **2.72** | 2.72 | 2.72 | **2.72** | 47,764 | **2.72** | 2.72 | 2.72 | **2.72** | 45,218 |
| 10081 | 300 | *Template-based* | **6.07** | 6.21 | 6.19 | 6.16 | 60,261 | 5.70 | 5.07 | **5.00** | 5.26 | 41,569 |
|  |  | *Deep Picker* | **7.15** | 7.42 | 7.22 | 7.26 | 42,374 | 6.15 | 6.02 | **5.99** | 6.05 | 28,209 |
|  |  | *CrYOLO* | **5.65** | 5.65 | 5.69 | 5.66 | 44,440 | **4.97** | 5.57 | 5.60 | 5.38 | 36,821 |
|  |  | *Topaz* | 6.29 | 6.32 | **6.13** | 6.25 | 58,217 | **5.08** | 5.13 | 5.09 | 5.10 | 37,808 |
|  |  | *CASSPER* | 6.47 | 6.39 | **6.20** | 6.35 | 39,724 | **5.75** | 5.84 | 5.78 | 5.79 | 27,299 |
|  |  | *CryoTransformer* | 5.58 | 5.61 | **5.53** | 5.57 | 112,119 | 5.52 | 5.45 | **5.44** | 5.47 | 88,632 |
|  |  | *CryoSegNet* | **4.58** | 4.59 | 4.62 | **4.60** | 60,158 | **4.16** | 4.17 | 4.20 | **4.18** | 44,819 |
| 10345 | 295 | *Template-based* | **6.00** | 6.46 | 6.50 | 6.32 | 26,727 | **4.03** | 4.03 | 4.15 | 4.07 | 14,353 |
|  |  | *Deep Picker* | 10.96 | **10.07** | 10.36 | 10.46 | 4,545 | **8.54** | 9.10 | 9.76 | 9.13 | 2,470 |
|  |  | *CrYOLO* | **3.98** | 3.98 | 4.30 | 4.09 | 15,821 | **3.83** | 3.98 | 4.00 | 3.94 | 11,369 |
|  |  | *Topaz* | 3.89 | 3.84 | **3.73** | 3.82 | 29,208 | **3.64** | 3.68 | 3.65 | 3.66 | 21,343 |
|  |  | *CASSPER* | 6.18 | **6.14** | 6.23 | 6.18 | 11,356 | **5.12** | 5.17 | 5.13 | 5.14 | 9,876 |
|  |  | *CryoTransformer* | 6.53 | 6.41 | **6.40** | 6.45 | 125,412 | 6.41 | **6.39** | 6.49 | 6.43 | 105,739 |
|  |  | *CryoSegNet* | **3.48** | 3.51 | 3.49 | **3.49** | 25,919 | 2.89 | **2.84** | 2.93 | **2.89** | 15,209 |
| 11056 | 305 | *Template-based* | 8.54 | 8.88 | **8.45** | 8.62 | 92,799 | **7.81** | 8.21 | 8.33 | 8.12 | 53,190 |
|  |  | *Deep Picker* | **9.92** | 11.40 | 10.47 | 10.60 | 21,005 | 9.79 | **9.54** | 9.62 | 9.65 | 17,124 |
|  |  | *CrYOLO* | 9.22 | 9.19 | **8.98** | 9.13 | 60,648 | 8.65 | **8.32** | 8.65 | 8.54 | 43,599 |
|  |  | *Topaz* | 8.23 | **8.11** | 8.18 | 8.17 | 98,680 | **8.03** | 8.06 | 8.10 | 8.06 | 66,651 |
|  |  | *CASSPER* | **9.19** | 9.28 | 9.21 | 9.23 | 52,143 | 8.46 | **8.42** | 8.53 | 8.47 | 34,860 |
|  |  | *CryoTransformer* | 8.09 | 8.13 | **8.06** | 8.09 | 152,768 | **7.39** | 7.46 | 7.41 | 7.42 | 98,193 |
|  |  | *CryoSegNet* | 7.88 | **7.83** | 7.92 | **7.88** | 71,342 | 7.21 | **7.13** | 7.16 | **7.17** | 53,073 |
| 10532 | 300 | *Template-based* | **4.02** | 4.03 | 4.02 | 4.02 | 90,262 | 3.93 | 3.93 | **3.92** | 3.93 | 43,662 |
|  |  | *Deep Picker* | 5.13 | **5.08** | 5.11 | 5.11 | 36,298 | 4.96 | **4.88** | 4.91 | 4.92 | 28,711 |
|  |  | *CrYOLO* | **4.23** | 4.29 | 4.24 | 4.25 | 46,162 | 4.12 | **4.08** | 4.10 | 4.10 | 29,434 |
|  |  | *Topaz* | 4.63 | **4.54** | 4.67 | 4.61 | 73,196 | **4.23** | 4.31 | 4.26 | 4.27 | 38,372 |
|  |  | *CASSPER* | 4.32 | **4.28** | 4.31 | 4.30 | 89,377 | 3.99 | **3.94** | 3.95 | 3.96 | 29,290 |
|  |  | *CryoTransformer* | 4.23 | 4.21 | **4.13** | 4.19 | 210,763 | **3.96** | 3.91 | 3.89 | **3.92** | 148,345 |
|  |  | *CryoSegNet* | 4.13 | 4.16 | **4.09** | **4.13** | 67,219 | 3.95 | **3.89** | 3.93 | **3.92** | 30,155 |
| 10093 | 295 | *Template-based* | **6.03** | 6.21 | 6.20 | **6.15** | 91,393 | 5.93 | **5.78** | 5.83 | **5.85** | 42,986 |
|  |  | *Deep Picker* | **10.69** | 10.98 | 10.97 | 10.88 | 3,193 | 7.69 | 7.56 | **7.25** | 7.50 | 2,360 |
|  |  | *CrYOLO* | 7.36 | **7.27** | 7.47 | 7.37 | 43,305 | **6.87** | 6.91 | 7.01 | 6.93 | 33,183 |
|  |  | *Topaz* | **6.35** | 6.51 | 6.42 | 6.43 | 110,577 | **6.12** | 6.18 | 6.14 | 6.15 | 61,698 |
|  |  | *CASSPER* | 8.77 | **8.68** | 8.73 | 8.73 | 41,638 | 7.26 | **7.23** | 7.32 | 7.27 | 32,383 |
|  |  | *CryoTransformer* | 7.23 | **7.18** | 7.25 | 7.22 | 230,390 | **6.81** | 6.95 | 6.82 | 6.86 | 151,545 |
|  |  | *CryoSegNet* | 7.33 | 7.42 | **7.27** | 7.34 | 43,886 | **6.99** | 7.17 | 7.01 | 7.06 | 27,745 |
| 10017 | 84 | *Template-based* | **5.06** | 5.15 | 5.13 | 5.11 | 63,301 | **4.96** | 5.00 | 5.04 | 5.00 | 49,770 |
|  |  | *Deep Picker* | 6.50 | **6.56** | 6.41 | 6.49 | 41,583 | 5.69 | 5.62 | **5.59** | 5.63 | 23,462 |
|  |  | *CrYOLO* | 5.14 | **4.99** | 5.11 | **5.08** | 54,263 | 4.87 | **4.84** | 4.89 | **4.87** | 47,704 |
|  |  | *Topaz* | **5.13** | 5.18 | 5.21 | 5.17 | 52,875 | **5.08** | 5.11 | 5.09 | 5.09 | 45,511 |
|  |  | *CASSPER* | **5.38** | 5.41 | 5.44 | 5.41 | 44,213 | **5.32** | 5.33 | 5.35 | 5.33 | 38,460 |
|  |  | *CryoTransformer* | **6.13** | 6.18 | 6.15 | 6.15 | 57,470 | **5.57** | 5.64 | 5.62 | 5.61 | 43,735 |
|  |  | *CryoSegNet* | 6.91 | **6.90** | 6.99 | 6.93 | 11,961 | 6.96 | 6.90 | **6.86** | 6.91 | 10,026 |

**Supplementary Table S3:** Comparison of 3D resolution of CryoSegNet on full set of micrographs with template-based picking, Deep Picker, crYOLO, Topaz, CASSPER and CryoTransformer. Bold font denotes the best resolution across 3 trials and bold underlined font represents the best average resolution across all methods.

| **EMPIAR ID** | **Number of Micrographs** | **Method** | **Without Select 2D** | | | | | **With Select 2D** | | | | | **Original EMPIAR** | |
| --- | --- | --- | --- | --- | --- | --- | --- | --- | --- | --- | --- | --- | --- | --- |
|  |  |  | **Resolution for 3 Trials (Å)** | | | **Average Resolution (Å)** | **Number of Particles** | **Resolution for 3 Trials (Å)** | | | **Average Resolution (Å)** | **Number of Particles** | **Resolution (Å)** | **Number of Particles** |
|  |  |  | **1** | **2** | **3** |  |  | **1** | **2** | **3** |  |  |  |  |
| 10028 | 600 | *Template-based* | 3.99 | 4.00 | **3.98** | 3.99 | 68,936 | 3.98 | **3.97** | 3.97 | 3.97 | 60,901 | 3.2 | 105,247 |
|  |  | *Deep Picker* | 4.11 | 4.11 | **4.10** | 4.11 | 50,451 | 4.09 | **4.09** | 4.10 | 4.09 | 43,027 |  |  |
|  |  | *CrYOLO* | 3.99 | 4.00 | **3.97** | 3.99 | 65,376 | 3.99 | **3.94** | 3.96 | 3.96 | 63,562 |  |  |
|  |  | *Topaz* | **2.72** | 2.72 | 2.72 | **2.72** | 104,652 | **2.72** | 2.72 | 2.72 | **2.72** | 96,352 |  |  |
|  |  | *CASSPER* | **4.23** | 4.20 | 4.21 | 4.21 | 32,570 | **4.16** | 4.17 | 4.19 | 4.17 | 29,906 |  |  |
|  |  | *CryoTransformer* | 4.02 | **3.99** | 3.99 | 4.00 | 99,767 | 3.76 | **3.72** | 3.73 | 3.74 | 81,172 |  |  |
|  |  | *CryoSegNet* | **2.72** | 2.72 | 2.72 | **2.72** | 93,881 | **2.72** | 2.72 | 2.72 | **2.72** | 92,532 |  |  |
| 10345 | 1644 | *Template-based* | 3.64 | **3.55** | 3.62 | 3.60 | 99,277 | 3.53 | 3.51 | **3.48** | 3.51 | 78,835 | 3.51 | 84,266 |
|  |  | *Deep Picker* | 7.21 | 7.30 | **7.10** | 7.20 | 18,590 | **4.16** | 4.21 | 4.25 | 4.21 | 8,399 |  |  |
|  |  | *CrYOLO* | **3.56** | 3.65 | 3.67 | 3.63 | 50,506 | **3.54** | 3.54 | 3.55 | 3.54 | 40,047 |  |  |
|  |  | *Topaz* | **3.50** | 3.55 | 3.52 | 3.52 | 102,977 | 3.48 | 3.46 | **3.45** | 3.46 | 87,472 |  |  |
|  |  | *CASSPER* | 5.68 | **5.59** | 5.63 | 5.63 | 68,217 | 4.03 | 4.07 | **3.99** | 4.03 | 56,728 |  |  |
|  |  | *CryoTransformer* | **5.22** | 5.39 | 5.25 | 5.29 | 182,397 | 3.51 | **3.45** | 3.48 | 3.48 | 111,375 |  |  |
|  |  | *CryoSegNet* | 2.79 | **2.74** | 2.75 | **2.76** | 120,357 | **2.67** | 2.70 | 2.69 | **2.69** | 73,377 |  |  |
| 10081 | 997 | *Template-based* | **4.32** | 4.36 | 4.35 | 4.34 | 177,647 | **4.09** | 4.20 | 4.10 | 4.13 | 134,603 | 3.5 | 55,870 |
|  |  | *Deep Picker* | 4.67 | **4.57** | 4.68 | 4.64 | 147,171 | 4.35 | 4.33 | **4.32** | 4.33 | 96,322 |  |  |
|  |  | *CrYOLO* | 4.32 | 4.28 | **4.26** | 4.29 | 148,488 | 4.20 | 4.19 | **4.15** | 4.18 | 123,963 |  |  |
|  |  | *Topaz* | **4.34** | 4.34 | 4.39 | 4.36 | 171,396 | **4.06** | 4.11 | 4.08 | 4.08 | 130,941 |  |  |
|  |  | *CASSPER* | 4.54 | **4.37** | 4.47 | 4.46 | 133,366 | **4.18** | 4.21 | 4.19 | 4.19 | 115,297 |  |  |
|  |  | *CryoTransformer* | 5.66 | 5.37 | **4.89** | 5.31 | 293,980 | 4.21 | 4.17 | **4.15** | 4.18 | 147,662 |  |  |
|  |  | *CryoSegNet* | 3.98 | **3.95** | 4.03 | **3.99** | 202,988 | 3.51 | **3.45** | 3.47 | **3.48** | 153,333 |  |  |
| 10532 | 1556 | *Template-based* | 3.79 | **3.72** | 3.85 | 3.79 | 604,356 | 3.28 | 3.29 | **3.25** | 3.27 | 234,512 | 2.9 | 128,305 |
|  |  | *Deep Picker* | 3.91 | 3.94 | **3.88** | 3.91 | 176,381 | **3.42** | 3.48 | 3.46 | 3.45 | 95,469 |  |  |
|  |  | *CrYOLO* | 3.26 | **3.25** | 3.28 | **3.26** | 232,220 | **3.22** | 3.25 | 3.22 | 3.23 | 161,497 |  |  |
|  |  | *Topaz* | 3.63 | 3.61 | **3.52** | 3.59 | 362,115 | 3.23 | **3.22** | 3.23 | 3.23 | 206,460 |  |  |
|  |  | *CASSPER* | 3.88 | 3.81 | **3.67** | 3.79 | 467,479 | 3.38 | **3.27** | 3.29 | 3.31 | 146,022 |  |  |
|  |  | *CryoTransformer* | **3.86** | 3.87 | 3.89 | 3.87 | 764,215 | **3.21** | 3.23 | 3.35 | 3.26 | 259,757 |  |  |
|  |  | *CryoSegNet* | 3.47 | **3.42** | 3.47 | 3.45 | 181,259 | 3.21 | 3.22 | **3.20** | **3.21** | 90,477 |  |  |
| 10093 | 1873 | *Template-based* | 4.45 | 4.42 | **4.34** | **4.40** | 645,308 | **4.10** | 4.15 | 4.13 | **4.13** | 391,973 | 3.55 | 175,314 |
|  |  | *Deep Picker* | **8.46** | 8.50 | 8.55 | 8.50 | 20,296 | 7.97 | **7.34** | 7.92 | 7.74 | 15,725 |  |  |
|  |  | *CrYOLO* | 4.61 | **4.54** | 4.58 | 4.58 | 264,447 | **4.41** | 4.42 | 4.46 | 4.43 | 192,337 |  |  |
|  |  | *Topaz* | 4.63 | **4.55** | 4.58 | 4.59 | 801,208 | 4.42 | **4.40** | 4.43 | 4.42 | 437,235 |  |  |
|  |  | *CASSPER* | 6.31 | **6.29** | 6.35 | 6.32 | 212,387 | **5.10** | 5.17 | 5.11 | 5.13 | 156,945 |  |  |
|  |  | *CryoTransformer* | 6.22 | 6.20 | **6.11** | 6.18 | 596,192 | 5.03 | 5.01 | **4.65** | 4.90 | 204,355 |  |  |
|  |  | *CryoSegNet* | 4.90 | **4.70** | 4.76 | 4.79 | 267,983 | 4.58 | **4.54** | 4.61 | 4.58 | 169,330 |  |  |

**Supplementary Table S4:** Comparative analysis of 3D resolution of CryoSegNet between the complete EMPIAR micrograph set and the smaller CryoPPP test dataset. Bold font denotes the highest resolution.

| **EMPIAR ID** | **CryoPPP Dataset** | | | **EMPIAR Dataset** | | |
| --- | --- | --- | --- | --- | --- | --- |
|  | **Number of Micrographs** | **Number of Particles** | **Best Resolution (Å)** | **Number of Micrographs** | **Number of Particles** | **Best Resolution (Å)** |
| 10028 | 300 | 47,764 | **2.72** | 600 | 92,532 | **2.72** |
| 10345 | 295 | 25,919 | 2.84 | 1,644 | 73,377 | **2.67** |
| 10081 | 300 | 60,158 | 4.16 | 997 | 153,333 | **3.45** |
| 10532 | 300 | 67,219 | 3.89 | 1,556 | 90,477 | **3.20** |
| 10093 | 295 | 43,886 | 6.99 | 1,873 | 169,330 | **4.54** |

**Supplementary Table S5:** Comparative analysis of 3D resolution of two small proteins between pre-trained CryoSegNet and fine-tuned CryoSegNet using predicted labels from pre-trained CryoSegNet. Bold font represents the highest resolution.

|  |  | **Pre-trained CryoSegNet** | | | | **Fine-tuned CryoSegNet** | | | |
| --- | --- | --- | --- | --- | --- | --- | --- | --- | --- |
|  |  |  |  |  |  |  |  |  |  |
| **EMPIAR ID** | **Number of Micrographs** | **Number of Particles** | **Average Resolution Å (Without Select 2D)** | **Number of Particles** | **Average Resolution Å (With Select 2D)** | **Number of Particles** | **Average Resolution Å (Without Select 2D)** | **Number of Particles** | **Average Resolution Å (With Select 2D)** |
|  |  |  |  |  |  |  |  |  |  |
| 11056 | 305 | 71,342 | 7.88 | 53,073 | 7.17 | 115,982 | 7 | 75,303 | **6.13** |
| 10017 | 84 | 11,961 | 6.93 | 10,026 | 6.91 | 38,349 | 5.52 | 33,572 | **5.27** |
| Average | 195 | 41,651 | 7.41 | 31,550 | 7.04 | 77,165 | 6.12 | 54,437 | **5.70** |

**Supplementary Table S6:** Carbon-alpha match score comparison of the 3D structures determined from the density maps of the particle picked by different methods with respect to the ground truth structure.

| **EMPIAR ID** | **Template-based** | **Deep Picker** | **CrYOLO** | **Topaz** | **CASSPER** | **CryoTransformer** | **CryoSegNet** |
| --- | --- | --- | --- | --- | --- | --- | --- |
| 10345 | 18.90 | 22.40 | 18.00 | 21.40 | 5.40 | 19.60 | **23.20** |
| 10081 | 23.20 | 22.40 | 21.70 | 21.90 | 15.70 | 22.70 | **26.00** |
| 10532 | 17.60 | 13.20 | **20.30** | 17.30 | 12.70 | 18.20 | 16.40 |
| 10093 | **18.30** | 3.40 | 17.00 | 13.40 | 5.40 | 13.60 | 12.70 |
| Average | 19.50 | 15.35 | 19.25 | 18.50 | 9.80 | 18.53 | **19.58** |

**Supplementary Table S7:** Performance of CryoSegNet over using non-denoised micrographs versus denoised micrographs with 4 encoder-decoder blocks. The use of denoised micrographs yields better performance.

| **Micrographs Type** | **Train Loss** | **Validation Loss** | **Train Dice Score** | **Validation Dice Score** | **Epochs Trained** |
| --- | --- | --- | --- | --- | --- |
| Non-denoised | 0.427 | 0.5182 | 0.587 | 0.4818 | 50 |
| Denoised | 0.4519 | 0.4341 | 0.5571 | 0.5689 | 50 |

**Supplementary Table S8:** Performance of CryoSegNet over using different number of encoder-decoder blocks with denoised micrographs. The use of 5 encoder-decoder blocks yields better performance.

| **Number of Encoder-Decoder** | **Train Loss** | **Validation Loss** | **Train Dice Score** | **Validation Dice Score** | **Epochs Trained** |
| --- | --- | --- | --- | --- | --- |
| 4 | 0.4519 | 0.4341 | 0.5571 | 0.5689 | 50 |
| 5 | 0.3896 | 0.3647 | 0.6275 | 0.6353 | 50 |
| 6 | 0.3942 | 0.3782 | 0.6158 | 0.6218 | 50 |

**Supplementary Table S9:** Performance of CryoSegNet over using different loss functions with 5 encoder-decoder blocks and denoised micrographs. The use of combined binary cross entropy and Dice loss yields better performance.

| **Loss Function** | **Train Loss** | **Validation Loss** | **Train Dice Score** | **Validation Dice Score** | **Epochs Trained** |
| --- | --- | --- | --- | --- | --- |
| Binary Cross Entropy (BCE) | 0.3896 | 0.3647 | 0.6275 | 0.6353 | 50 |
| Dice Loss | 0.3433 | 0.3419 | 0.6728 | 0.6565 | 50 |
| BCE Loss + Dice Loss | 0.3484 | 0.3219 | 0.6704 | 0.6781 | 50 |

**Supplementary Table S10:** Performance of CryoSegNet over using attention-gate with 5 encoder-decoder blocks, combined BCE loss and Dice loss and denoised micrographs. The use of attention-gate yields better performance over not using it.

| **Use of Attention-gate** | **Train Loss** | **Validation Loss** | **Train Dice Score** | **Validation Dice Score** | **Epochs Trained** |
| --- | --- | --- | --- | --- | --- |
| No | 0.3484 | 0.3219 | 0.6704 | 0.6781 | 50 |
| Yes | 0.3189 | 0.303 | 0.6997 | 0.697 | 50 |

**Supplementary Table S11:** Performance of CryoSegNet over different shapes of input micrographs with 5 encoder-decoder blocks, combined BCE loss and Dice loss, attention-gate and denoised micrographs. The use of 1024 x 1024 shaped micrograph yields better performance.

| **Shape of Input Micrograph** | **Train Loss** | **Validation Loss** | **Train Dice Score** | **Validation Dice Score** | **Epochs Trained** |
| --- | --- | --- | --- | --- | --- |
| 512 x 512 | 0.417 | 0.4576 | 0.5962 | 0.5424 | 50 |
| 1024 x 1024 | 0.3189 | 0.303 | 0.6997 | 0.697 | 50 |
| 2048 x 2048 | 0.103 | 0.8404 | 0.9077 | 0.2642 | 50 |

**Supplementary Table S12**: An overview of the dataset used for training and validation of CryoSegNet (* represents theoretical weight of the proteins)

| **SN** | **EMPIAR ID** | **Type of Protein** | **Image Size** | **Total Structure Weight (kDa)** | **Training Images** | **Validation Images** | **Total Images** |
| --- | --- | --- | --- | --- | --- | --- | --- |
| 1 | 10005 [10] | TRPV1 Transport Protein | (3710, 3710) | 272.97 | 23 | 6 | 29 |
| 2 | 10059 [11] | TRPV1 Transport Protein | (3838, 3710) | 317.88 | 232 | 59 | 291 |
| 3 | 10075 [12] | Bacteriophage MS2 | (4096, 4096) | 1000* | 239 | 60 | 299 |
| 4 | 10077 [13] | Ribosome (70S) | (4096, 4096) | 2198.78 | 240 | 60 | 300 |
| 5 | 10096 [14] | Viral Protein | (3838, 3710) | 150* | 240 | 60 | 300 |
| 6 | 10184 [15] | Aldolase | (3838, 3710) | 150* | 236 | 60 | 296 |
| 7 | 10240 [16] | Lipid Transport Protein | (3838, 3710) | 171.72 | 239 | 60 | 299 |
| 8 | 10289 [17] | Transport Protein | (3710, 3838) | 361.39 | 240 | 60 | 300 |
| 9 | 10291 [17] | Transport Protein | (3710, 3838) | 361.39 | 240 | 60 | 300 |
| 10 | 10387 [18] | Viral Protein | (3710, 3838) | 185.87 | 239 | 60 | 299 |
| 11 | 10406 [19] | Ribosome (70S) | (3838, 3710) | 632.89 | 191 | 48 | 139 |
| 12 | 10444 [20] | Membrane Protein | (5760, 4092) | 295.89 | 236 | 60 | 296 |
| 13 | 10526 [21] | Ribosome (50S) | (7676, 7420) | 1085.81 | 176 | 44 | 220 |
| 14 | 10590 [22] | TRPV1 Transport Protein | (3710, 3838) | 1000* | 236 | 60 | 296 |
| 15 | 10671 [23] | Signaling Protein | (5760, 4092) | 77.14 | 238 | 60 | 298 |
| 16 | 10737 [24] | Membrane Protein | (5760, 4092) | 155.83 | 233 | 59 | 292 |
| 17 | 10760 [25] | Membrane Protein | (3838, 3710) | 321.69 | 240 | 60 | 300 |
| 18 | 10816 [26] | Transport Protein | (7676, 7420) | 166.62 | 240 | 60 | 300 |
| 19 | 10852 [27] | Signaling Protein | (5760, 4092) | 157.81 | 274 | 69 | 343 |
| 20 | 11051 [28] | Transcription/DNA/RNA | (3838, 3710) | 357.31 | 240 | 60 | 300 |
| 21 | 11057 [29] | Hydrolase | (5760, 4092) | 149.43 | 236 | 59 | 295 |
| 22 | 11183 [30] | Signaling Protein | (5760, 4092) | 139.36 | 240 | 60 | 300 |
| Total | | | | | 4,948 | 1,244 | 6,192 |

**Supplementary Table S13:** An overview of the independent dataset for testing CryoSegNet (* represents theoretical weight of the proteins)

| **SN** | **EMPIAR ID** | **Type of Protein** | **Image Size** | **Total Structure Weight (kDa)** | **Number of Images** |
| --- | --- | --- | --- | --- | --- |
| 1 | 10028 [4] | Ribosome (80S) | (4096, 4096) | 2135.89 | 300 |
| 2 | 10081 [7] | Transport Protein | (3710, 3838) | 298.57 | 300 |
| 3 | 10345 [31] | Signaling Protein | (3838, 3710) | 244.68 | 295 |
| 4 | 11056 [32] | Transport Protein | (5760, 4092) | 88.94 | 305 |
| 5 | 10532 [33] | Viral Protein | (4096, 4096) | 191.76 | 300 |
| 6 | 10093 [5] | Membrane Protein | (3838, 3710) | 779.4 | 295 |
| 7 | 10017 [6] | β -galactosidase | (4096, 4096) | 450* | 84 |
| Total | | | | | 1,879 |

**Supplementary Table S14**: Topaz false positive filtering using different log-likelihood thresholds

| EMPIAR ID | Number of Micrographs | Particle Threshold Parameter = 0 | | Particle Threshold Parameter = 1 | | Particle Threshold Parameter = 2 | |
| --- | --- | --- | --- | --- | --- | --- | --- |
|  |  | Best Resolution (Å) | Number of Particles | Best Resolution (Å) | Number of Particles | Best Resolution (Å) | Number of Particles |
| 10028 | 600 | **2.72** | 273,432 | 2.72 | 165,685 | 2.72 | 108,709 |
| 10345 | 1644 | **3.46** | 245,255 | 3.47 | 170,469 | 3.48 | 83,890 |
| 10081 | 997 | 4.19 | 215,631 | **4.16** | 153,209 | 4.24 | 143,437 |
| 10532 | 1556 | **3.27** | 371,285 | 3.34 | 293,112 | 3.33 | 222,685 |
| 10093 | 1873 | **4.37** | 597,601 | 4.48 | 467,788 | 4.59 | 206,796 |

***Supplementary Algorithm S1: Postprocessing of the output of SAM***

**Require:** a segmentation mask from SAM’s automatic mask generator as input.

1. Consider only the particles with a predicted IoU greater than 0.94.
2. Extract the bounding-box information ‘bbox’ for each picked particle in the segmentation mask, where the 1^st^ and 2^nd^ values are the x and y coordinates, and the 3^rd^ and 4^th^ values are the width and height, respectively.
3. Calculate the mode of the widths (m_w) and mode of the heights (m_h) for the particles from step 2 for each segmentation mask.
4. Determine the new diameter (d) of the picked particles from each segmentation mask. Rescale the m_w and m_h values from step 3 according to the size of original micrograph. Calculate d using the formula:

$d =\sqrt{\left\{ \left( m\_w \cdot\frac{o\_w}{1024} \right) \right\}^{2} +\left\{ \left( m\_h \cdot\frac{o\_h}{1024} \right) \right\}^{2}}$ (1)

where, o_w and o_h are the width and height of the original micrograph.

1. Set a threshold value (th) equal to 10% of the diameter:

$th = 0.1 \cdot d$ (2)

1. Select particles with width and height that satisfy the following criteria:

$m\_w-\frac{th}{3} <width<m\_w+th$ (3)

$m\_h-\frac{th}{3} <height<m\_h+th$ (4)

1. Calculate the scaled x and y-coordinates of the center of the protein particles for each segmentation mask of micrograph:

$new\_x = \left( \frac{x-coordinate +\frac{width}{2}}{1024} \right)\cdot width$ (5)

$new\_y = \left( \frac{y-coordinate +\frac{height}{2}}{1024} \right)\cdot height$ (6)

1. Output the values new_x, new_y and d of each particle from micrographs to a .star file.

References

1. Wagner T, Merino F, Stabrin M, et al. SPHIRE-crYOLO is a fast and accurate fully automated particle picker for cryo-EM. Commun Biol 2019; 2:
2. Bepler T, Morin A, Rapp M, et al. Positive-unlabeled convolutional neural networks for particle picking in cryo-electron micrographs. Nat Methods 2019; 16:1153–1160
3. Dhakal A, Gyawali R, Wang L, et al. A large expert-curated cryo-EM image dataset for machine learning protein particle picking. Sci Data 2023; 10:
4. Wong W, Bai XC, Brown A, et al. Cryo-EM structure of the Plasmodium falciparum 80S ribosome bound to the anti-protozoan drug emetine. Elife 2014; 2014:
5. Jin P, Bulkley D, Guo Y, et al. Electron cryo-microscopy structure of the mechanotransduction channel NOMPC. Nature 2017; 547:118–122
6. Scheres SHW. Semi-automated selection of cryo-EM particles in RELION-1.3. J Struct Biol 2015; 189:114–122
7. Lee CH, MacKinnon R. Structures of the Human HCN1 Hyperpolarization-Activated Channel. Cell 2017; 168:111-120.e11
8. AU - Moriya T, AU - Saur M, AU - Stabrin M, et al. High-resolution Single Particle Analysis from Electron Cryo-microscopy Images Using SPHIRE. JoVE 2017; e55448
9. Kirillov A, Mintun E, Ravi N, et al. Segment Anything. arXiv:2304.02643 2023;
10. Liao M, Cao E, Julius D, et al. Structure of the TRPV1 ion channel determined by electron cryo-microscopy. Nature 2013; 504:107–112
11. Gao Y, Cao E, Julius D, et al. TRPV1 structures in nanodiscs reveal mechanisms of ligand and lipid action. Nature 2016; 534:347–351
12. Koning RI, Gomez-Blanco J, Akopjana I, et al. Asymmetric cryo-EM reconstruction of phage MS2 reveals genome structure in situ. Nat Commun 2016; 7:
13. Fischer N, Neumann P, Bock L V., et al. The pathway to GTPase activation of elongation factor SelB on the ribosome. Nature 2016; 540:80–85
14. Zi Tan Y, Baldwin PR, Davis JH, et al. Addressing preferred specimen orientation in single-particle cryo-EMthrough tilting. Nat Methods 2017; 14:793–796
15. Kim LY, Rice WJ, Eng ET, et al. Benchmarking cryo-EM single particle analysis workflow. Front Mol Biosci 2018; 5:
16. Falzone ME, Rheinberger J, Lee BC, et al. Structural basis of ca2+-dependent activation and lipid transport by a tmem16 scramblase. Elife 2019; 8:
17. Burendei B, Shinozaki R, Watanabe M, et al. Cryo-EM structures of undocked innexin-6 hemichannels in phospholipids. Sci. Adv 2020; 6:
18. Passos DO, Li M, Jóźwik IK, et al. Structural basis for strand-transfer inhibitor binding to HIV intasomes. Science (1979) 2020; 367:810–814
19. Nicholson D, Edwards TA, O’Neill AJ, et al. Structure of the 70S Ribosome from the Human Pathogen Acinetobacter baumannii in Complex with Clinically Relevant Antibiotics. Structure 2020; 28:1087-1100.e3
20. Demura K, Kusakizako T, Shihoya W, et al. Cryo-EM structures of calcium homeostasis modulator channels in diverse oligomeric assemblies. Sci. Adv 2020; 6:
21. Li Q, Pellegrino J, Lee DJ, et al. Synthetic group A streptogramin antibiotics that overcome Vat resistance. Nature 2020; 586:145–150
22. Mashtalir N, Suzuki H, Farrell DP, et al. A Structural Model of the Endogenous Human BAF Complex Informs Disease Mechanisms. Cell 2020; 183:802-817.e24
23. Josephs TM, Belousoff MJ, Liang YL, et al. Structure and dynamics of the CGRP receptor in apo and peptide-bound forms. Science (1979) 2021; 372:
24. Li J, Han L, Vallese F, et al. Cryo-EM structures of Escherichia coli cytochrome bo3 reveal bound phospholipids and ubiquinone-8 in a dynamic substrate binding site. Proceedings of the National Academy of Sciences 2021; 118:e2106750118
25. Kuzuya M, Hirano H, Hayashida K, et al. Structures of human pannexin-1 in nanodiscs reveal gating mediated by dynamic movement of the N terminus and phospholipids. Sci. Signal 2022; 15:
26. Oldham ML, Grigorieff N, Chen J. Structure of the transporter associated with antigen processing trapped by herpes simplex virus. Elife 2016; 5:e21829
27. Cao C, Kang HJ, Singh I, et al. Structure, function and pharmacology of human itch GPCRs. Nature 2021; 600:170–175
28. Newing TP, Oakley AJ, Miller M, et al. Molecular basis for RNA polymerase-dependent transcription complex recycling by the helicase-like motor protein HelD. Nat Commun 2020; 11:
29. Tanaka S, Morita M, Yamagishi T, et al. Structural Basis for Binding of Potassium-Competitive Acid Blockers to the Gastric Proton Pump. J Med Chem 2022; 65:7843–7853
30. Liu Y, Cao C, Huang XP, et al. Ligand recognition and allosteric modulation of the human MRGPRX1 receptor. Nat Chem Biol 2023; 19:416–422
31. Campbell MG, Cormier A, Ito S, et al. Cryo-EM Reveals Integrin-Mediated TGF-β Activation without Release from Latent TGF-β. Cell 2020; 180:490-501.e16
32. Asami J, Kimura KT, Fujita-Fujiharu Y, et al. Structure of the bile acid transporter and HBV receptor NTCP. Nature 2022; 606:1021–1026
33. Tan YZ, Rubinstein J. Through-grid wicking enables high-speed cryoEM specimen preparation. Microscopy and Microanalysis 2021; 27:526–528
